# Insights into biological activity of ureidoamides with primaquine and amino acid moieties

**DOI:** 10.1080/14756366.2017.1423067

**Published:** 2018-01-24

**Authors:** Josipa Vlainić, Ivan Kosalec, Kristina Pavić, Dimitra Hadjipavlou-Litina, Eleni Pontiki, Branka Zorc

**Affiliations:** a Laboratory for Advanced Genomics, Division of Molecular Medicine, Rudjer Bošković Institute, Zagreb, Croatia;; b Faculty of Pharmacy and Biochemistry, Department of Microbiology, University of Zagreb, Zagreb, Croatia;; c Faculty of Pharmacy and Biochemistry, Department of Medicinal Chemistry, University of Zagreb, Zagreb, Croatia;; d School of Pharmacy, Faculty of Health Sciences, Aristotle University of Thessaloniki, Thessaloniki, Greece

**Keywords:** Primaquine, ureidoamide, biofilm eradication, antibacterial activity, antioxidative screening

## Abstract

Primaquine (PQ) ureidoamides **5a–f** were screened for antimicrobial, biofilm eradication and antioxidative activities. Susceptibility of the tested microbial species towards tested compounds showed species- and compound-dependent activity. *N*-(diphenylmethyl)-2-[({4-[(6-methoxyquinolin-8-yl)amino]pentyl}carbamoyl)amino]-4-methylpentanamide (**5a**) and 2-(4-chlorophenyl)-*N*-(diphenylmethyl)-2-[({4-[(6-methoxyquinolin-8-yl)amino]pentyl}carbamoyl)amino]acetamide (**5d**) showed antibacterial activity against *S. aureus* strains (MIC = 6.5 µg/ml). Further, compounds **5c** and **5d** had weak antibacterial activity against *Escherichia coli* and *Pseudomonas aeruginosa*. None of the tested compounds showed a wide spectrum of antifungal activity. In contrast, most of the compounds exerted strong activity in a biofilm eradication assay against *E. coli*, *P. aeruginosa* and *Candida albicans*, comparable to or even higher than gentamycin, amphotericin B or parent PQ. The most active compounds were **5a** and **5b**. Tested compounds were inactive against biofilm formation by *C. parapsylosis*, *Enterococcus faecalis, C. tropicalis* and *C. krusei.* Compounds **5b–f** significantly inhibited lipid peroxidation (80–99%), whereas compound **5c** presented interesting LOX inhibition.

## Introduction

Primaquine (8-[(4-amino-1-methylbutyl)amino]-6-methoxyquinoline, PQ) is a synthetic antimalarial drug that eliminates exoerythrocytic infection. It prevents the development of dormant liver forms of the parasite, which are responsible for relapses in vivax and ovale malaria. PQ is also active against gametocytes of all parasite species causing human malaria, including chloroquine-resistant *Plasmodium falciparum*^1^. In addition, PQ and other antimalarial drugs (artemisinin, artesunate, tetracyclines and chloroquine) show significant anticancer activity, reduce IC_50_ of anticancer drugs, affect their ADME properties leading to improvement of tumour therapy or inhibit development of drug-resistant cancer cells. Numerous reports corroborate anticancer properties of antimalarial drug classes and their usefulness in adjuvant chemotherapies^2–6^.

Our scientific efforts are directed towards new anticancer agents derived from PQ. In the last few years we have designed, prepared and biologically evaluated approximately a hundred PQ-derivatives of amide, urea, *bis*-urea, hydroxyurea, semicarbazide and acylsemicarbazide type. Many of them have shown a strong antiproliferative effect against a number of tumour cell lines and/or significant antioxidative properties^7–12^. In addition, PQ-cinnamamide, PQ-*bis*-trifluoromethylated cinnamamide and PQ-urea derivatives with hydroxyl, halogen and trifluoromethyl substituents on the benzene ring exerted very strong antimycobacterial activity towards three *Mycobacterium* species, stronger than PQ and the standard antitubercular drugs^13^. PQ-aryl urea derivative, in particular that with a trifluoromethyl moiety, exhibited significant antimicrobial activity with MIC values from 1.6 to 12.5 µg/ml^11^. Antimalarial activity of our compounds against the erythrocytic stage of drug-sensitive *Plasmodium falciparum* was evaluated as well. After detailed QSAR studies, a series of ureidoamides **5a–f** bearing PQ and amino acid moieties were designed, prepared and evaluated against the same parasite species^14^.

In order to get a better insight into the pharmacological potential of novel ureidoamides, their antimicrobial and antioxidative activities were evaluated and are reported herein. In addition, we were especially interested in their biofilm eradication potential. Biofilms are structured communities of microorganisms formed on biotic or abiotic (medical devices and biomaterials) surfaces, which are often responsible for therapy failures in clinics^15^. Biofilm formation starts with microbial attachment, its reproduction and micro-colony formation and leads to microorganism enclosure through self-formation of protective matrices (extracellular polymeric structures), which results in increased resistance to antimicrobial agents and host immune factors^16^. Thus, biofilm-associated infections are, in general, chronic infections with high morbidity and mortality rates^17^^,^^18^. Therefore, many efforts have been made to find drugs effective in inhibition of biofilm formation and its eradication^19^.

## Materials and methods

### Chemistry

#### General information

Melting points were measured on a Stuart Melting Point (SMP3) apparatus (Barloworld Scientific, UK) in open capillaries with uncorrected values. IR spectra were recorded on FTIR Spectrum One spectrophotometer (Perkin-Elmer, UK). NMR (^1^H and ^13^C) were recorded at 25 °C on NMR Avance 600 spectrometer (Bruker, Germany) at 300 and 150 MHz for ^1^H and ^13^C nuclei, respectively. All compounds were routinely checked by TLC with Merck silica gel 60 F-254 glass plates using the following solvent systems: petrolether/ethyl acetate/methanol 30:10:5, cyclohexane/ethyl acetate 1:1, cyclohexane/dichloromethane/methanol 10:18:2, dichloromethane/methanol 97:3 and 95:5. Spots were visualised by short-wave UV light and iodine vapour. Column chromatography was performed on silica gel 0.063–0.200 mm (Kemika, Croatia) and 0.040–0.063 mm (Merck, Germany), with the same eluents used in TLC. 1*H*-benzo[*d*][1,2,3]triazole (BtH), triphosgene, triethylamine (TEA), hydrazine hydrate, L-leucine, D-phenylglycine, DL-*p*-chlorophenylglycine and *N*-methyl-1,1-diphenylmethanamine were purchased from Sigma-Aldrich, USA. Primaquine, diphenylmethanamine and (4-chlorophenyl)(phenyl)methanamine were prepared from commercially available salts (Sigma-Aldrich) prior the use. All reactions with primaquine were run light protected.

#### Synthesis

Ureidoamides **5a–f** with primaquine and amino acid moieties were prepared according to a procedure developed by our research group^14^. Synthetic pathway leading to compounds **5a–f** included preparation of several precursors: 1-benzotriazole carboxylic acid chloride (benzotriazolecarbonyl chloride, BtcCl, **1**), Btc-amino acids **2a–c**, Btc-amino acid chlorides **3a–c** and Btc-amino acid amides **4a–f**. The following PQ-ureidoamides were prepared: *N*-(diphenylmethyl)-2-[({4-[(6-methoxyquinolin-8-yl)amino]pentyl}carbamoyl)amino]-4-methylpentanamide (**5a**), *N*-(diphenylmethyl)-2-[({4-[(6-methoxyquinolin-8-yl)amino]pentyl}carbamoyl)amino]-*N*-methyl-2-phenylacetamide (**5b**), *N*-[(4-chlorophenyl)(phenyl)methyl]-2-[({4-[(6-methoxyquinolin-8-yl)amino]pentyl}carbamoyl)amino]-2-phenylacetamide (**5c**), 2–(4-chlorophenyl)-*N*-(diphenylmethyl)-2-[({4-[(6-methoxyquinolin-8-yl)amino]pentyl}carbamoyl)amino]acetamide (**5d**), 2–(4-chlorophenyl)-*N*-[(4-chlorophenyl)(phenyl)methyl]-2-[({4-[(6-methoxyquinolin-8-yl)amino]pentyl}carbamoyl)amino]acetamide (**5e**) and 3-[(4-chlorophenyl)({*N*'-[(4-methoxyphenyl)(phenyl)methylidene]hydrazinecarbonyl})methyl]-1-{4-[(6-methoxyquinolin-8-yl)amino]pentyl}urea (**5f**). Their analytical and spectral data were fully in agreement with the previously obtained data.

### Biological screening

#### General information

Absorbances were measured on a Lambda 20 double-beam spectrophotometer (Perkin-Elmer, UK). Centrifuge 5810 R (Eppendorf, Germany), microplate reader (iEMS, Finland) and Multiscan plate reader (Thermo) were used. Linoleic acid sodium salt, soybean lipoxygenase (LOX), 1,1-diphenyl-2-picrylhydrazyl (DPPH), nordihydroguaiaretic acid (NDGA), 2,2’-azobis(2-amidinopropane) dihydrochloride (AAPH), 6-hydroxy-2,5,7,8-tetramethylchroman-2-carboxylic acid (Trolox), phosphate-buffered saline (PBS), Triton X100 and foetal bovine serum (FBS) were purchased from Sigma-Aldrich.

### *In vitro* antimicrobial susceptibility assay (MIC determination)

Before analysis, compounds **5a–f** were dissolved in 96% ethanol in a concentration of 2 mg/ml as stock solutions. Inoculums were prepared with fresh cultures of microbial strains, cultured on tryptic-soy agar or Sabouraud 2% (*m/v*) dextrose agar for 18 h (48–72 h for fungi) at 30 °C with physiological saline containing approximately 3 × 10^6^ CFUs/ml. Inoculum density (0.5 McFarland units) was adjusted with a bio Mériex (France) densitometer. Minimum inhibitory concentration (MIC) was determined by the twofold micro-dilution method in Mu¨ller–Hinton broth for bacterial strains and RPMI 1640 + 2% *m/v* dextrose for fungi according to the EUCAST recommendations in a sterile 96-well flat-bottom plastic tissue culture plate^20^. MIC was determined using a linear regression curve after incubation of compounds and reading the optical density at 570 nm using an iEMS microplate reader. MIC was defined as the lowest concentration of extract that allows no more than 50% growth of microbes in comparison with intact microbial cells (negative control). All tests were performed in triplicate and expressed as the mean.

### Minimum biofilm eradication assay

We tested the susceptibility of different bacterial and yeast strains to compounds **5a–f** by determining the minimum biofilm eradication concentration (MBEC). The test was performed in a sterile 96-well flat-bottom plastic tissue culture plate (TPP, Switzerland). Each well was filled with 100 µl of bacterial (10^7^ CFUs/ml) or yeast (5 × 10^6^ CFUs/ml) suspension. When the possibility of inhibition of yeast biofilm formation was tested, the wells were pre-treated with FBS (250 µl per well). Negative controls contained broth only. Positive controls were performed using standard antibacterial and antifungal drugs gentamycin and amphotericin B, respectively. The plates were covered and incubated aerobically for 24 h (bacteria) or 48 h (yeast) at 37 °C. Following the incubation period, each well was aspirated and washed three times with 250 µl of PBS and vigorously shaken in order to remove all non-adherent bacteria/yeast. The remaining attached cells were fixed with methanol (15 min) and the plates were left to dry overnight. The formed biofilm was stained with crystal violet (1%, 5 min). Excess stain was rinsed by placing the plate under running tap water and the plates were left to dry for 24 h. Adherent cells were resolubilized with acetic acid (33%, *v/v*). The optical density of each well was measured at 570 nm using a Multiscan plate reader. The MBEC value represents the lowest compound dilution at which microorganisms fail to grow.

### Antioxidative activity

#### Interaction of the PQ-ureidoamides 5a–f with 1,1-diphenyl-2-picrylhydrazyl (DPPH)

To a solution of DPPH in absolute ethanol, an appropriate volume of the tested compound solution (0.1 mM final concentration) dissolved in DMSO was added. Absorbance was recorded at 517 nm after 20 and 60 min at room temperature (Lambda 20 double beam spectrophotometer, Perkin-Elmer, UK). Experiments were repeated at least in triplicate and the standard deviation of absorbance was less than 10% of the mean^10^. NDGA was used under the same experimental conditions as a reference compound.

#### *In vitro* soybean lipoxygenase (LOX) inhibition study

*In vitro* LOX inhibition assay was accomplished as described previously^10^. Test compounds (stock solutions 10 mM in DMSO) were incubated at room temperature with sodium linoleate (0.1 mM) and 0.2 ml of LOX solution (0.1 mg in 10 ml saline). Conversion of sodium linoleate to 13-hydroperoxylinoleic acid was measured at 234 nm and compared to the reference inhibitor. Several concentrations were used for IC_50_ determination. The assays were repeated at least in triplicate and the standard deviation of absorbance was less than 10% of the mean. NDGA was used under the same experimental conditions as a reference compound.

#### Inhibition of linoleic acid peroxidation (LP)

To initiate lipid peroxidation, the free radical AAPH was used^10^. The final solution in the UV cuvette consisted of 10 µl sodium linoleate solution (*c* = 16 mM) and 0.93 ml phosphate buffer pH 7.4 (*c* = 50 mM). Afterwards, 10 µl of the tested compound solution in DMSO (final concentration 0.1 mM) and 50 µl of AAPH solution (*c* = 40 mM) were added. The experiment was performed at 37 °C under air. Oxidation of linoleic acid sodium salt was monitored at 234 nm. The assay was repeated at least in triplicate and the standard deviation of absorbance was less than 10% of the mean. Trolox was used under the same experimental conditions as a reference compound.

#### *In vitro* haemolytic activity

Whole venous human blood (10 ml) was collected in a heparin-based container from a volunteer donor (45 years old, non-smoker) after obtaining his consent and washed three times with PBS pH 7.0 (centrifugation at 2500 rpm, 4 min, Centrifuge 5810 R, Eppendorf, Germany). Then, 4% (*v/v*) of fresh human erythrocyte solution (hErc) was prepared using a PBS pH 7.0 solution. A series of diluted compounds from 200 to 50 µg/ml were prepared in a total volume of 1 ml with hErc solution. After incubation for 1 h at 37 °C aerobically (without shaking), test tubes were centrifuged at room temperature (2500 rpm, 4 min). Absorbance of the supernatant (200 µl) was recorded at 540 nm using an iEMS microplate reader. The 0.1% (*v/v*) solution of Triton X100 served as a positive control (as total lysis of hErc), and intact hErc served as a negative control. Since compounds were diluted with 1.5% (*v/v*) DMSO, the same solution was used as a control. All tests were performed in triplicate and the results were expressed as haemolysis percentage (mean ± SD). Percentage of haemolysis was calculated using the following equation:

$$haemolysis\;\left(\%\right)=\frac{A_{sample}-A_{nc}}{A_{pc}-A_{nc}}\times 100$$

where *A*_sample_ was absorbance of the tested compound, *A*_nc_ was absorbance of the negative control (intact hErc) and *A*_pc_ was absorbance of the positive control treated with 0.1% TritonX100. In statistical calculations, p values below 0.05 were considered significant throughout the test. For comparison of mean values, one-way ANOVA with Dunnet’s post-test was used.

## Results and discussion

### Chemistry

**Scheme 1.**
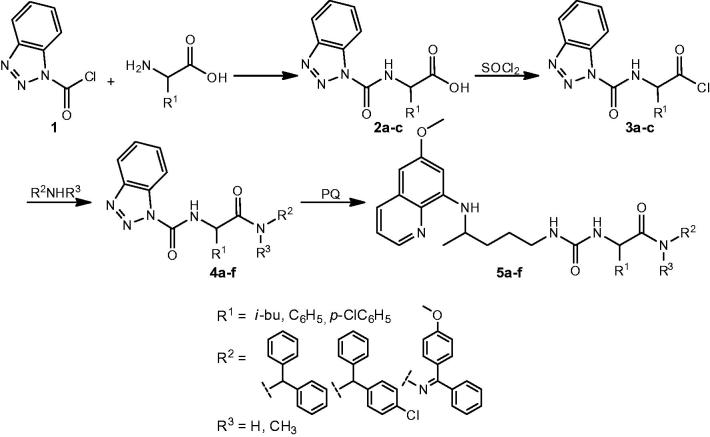
Synthesis of ureidoamides **5a–f** and their precursors.

Preparation of PQ-ureidoamides **5a–f** was complex and involved several reaction steps: preparation of Btc-amino acids **2a–c** from BtcCl **1** and the corresponding amino acid, conversion of Btc-amino acid to Btc-amino acid chloride **3a–c**, reaction of Btc-amino acid chlorides with amines or hydrazone and aminolysis of Btc-amino acid amides **4a–f** with primaquine (Scheme 1).

**Figure 1.**
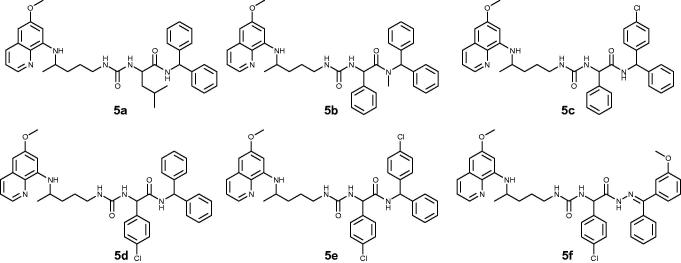
Chemical structures of PQ-ureidoamides **5a–f**.

Three amino acids (L-leucine, D-phenylglycine, DL-*p*-chlorophenylglycine), three amines (diphenylmethanamine, (4-chlorophenyl)(phenyl)methanamine)), *N*-methyl-1,1-diphenylmethanamine and one hydrazone (4-methoxybenzophenone hydrazone) were used. Structures of PQ-ureidoamides **5a–f** are given in Figure 1.

Compounds **5a–f** are not fully in agreement with Lipinski’s and Gelovani’s rules for prospective small molecular drugs but show acceptable deviations from the rules^21^. The parameters were calculated with the Chemicalize.org program^22^ and are presented in Table 1.

**Table 1.** Properties of PQ-ureidoamides **5a–f** calculated with Chemicalize.org programme:^22^ Lipinski's and Gelovani's parameters.

Compd.	Molecular formula	Number of atoms	MW	log *P*	H-bond donor	H-bond acceptor	Lipinski score^a^	MR (cm^3^/mol)	PSA (Å^2^)
**5a**	C_35_H_43_N_5_O_3_	86	581.76	5.58	4	5	2	171.40	104.38
**5b**	C_38_H_41_N_5_O_3_	87	615.78	5.92	3	5	2	182.49	95.59
**5c**	C_37_H_38_ClN_5_O_3_	84	636.19	6.30	4	5	2	182.39	104.38
**5d**	C_37_H_38_ClN_5_O_3_	84	636.18	6.30	4	5	2	182.39	104.38
**5e**	C_37_H_37_Cl_2_N_5_O_3_	84	670.64	6.90	4	5	2	187.20	104.38
**5f**	C_38_H_39_ClN_6_O_4_	88	679.22	6.29	4	7	2	192.16	125.97

^a^out of four; MR: molecular refractivity; PSA: polar surface area.

### Biological evaluation

Based on previous findings on the antimicrobial effects of PQ-derivatives, we tested PQ-ureidoamides **5a–f** toward several bacterial and fungal species. To assess this, we applied the broth dilution method to determine MIC of each compound towards a selected panel of microorganisms: Gram-positive bacteria (*Staphylococcus aureus, Enterococcus faecalis, Enterococcus hyrae, Kocuria rhizophila, Bacillus cereus, Bacillus subtilis*), Gram-negative bacteria (*Escherichia coli, Pseudomonas aeruginosa, Burkholderia caepacia*) and seven fungi (*Candida albicans, Candida kefyr, Candida krusei, Candida parapsylosis, Candida tropicalis, Issatchenkia orientalis* Kudrjanzev, *Aspergillus niger*). MICs of the tested compounds, together with positive controls (tetracycline and amphotericin B), were determined after 18- or 24-h incubation of the selected microbial species and the values are listed in Supplementary material.

Susceptibility of the tested microbial species towards PQ-derivatives **5a–f** showed species-dependent and compound-dependent activity. All MICs lower than 100 µg/ml were set as a pronounced antimicrobial activity. Only compounds **5a** and **5d** showed antibacterial activity against the tested *Staphylococcus* spp. with the lowest MIC value of 6.5 µg/ml (MIC range 6.5–15 µg/ml for **5a** and 12.5–25 µg/ml for **5d**). Compound **5a** showed weak activity towards *K. rhizophila* and *P. aeruginosa* (MIC = 25 µg/ml) and *B. subtilis* (MIC = 50 µg/ml). Furthermore, compounds **5c** and **5d** showed low antibacterial activity against two Gram-negative species (*E. coli* and *P. aeruginosa*, MIC range from 25 to 65 µg/ml). *E. coli* displayed a slight susceptibility towards compound **5e** as well (MIC = 45–60 µg/ml). None of the tested compounds showed a wide spectrum of antifungal activity against yeasts and filamentous fungi.

Modern medicine uses a broad variety of medical devices and implants, the application of which is often associated with attachment of microorganisms and biofilm formation. Microorganisms within a biofilm are more resistant to different antimicrobial treatments; they can survive even under harsh environmental conditions and withstand the host’s immune system^23^. Therefore, search for novel compounds that will combat biofilms at different levels of formation and maturation is extremely important. Since the PQ-derivatives **5a–f** showed, to some extent, antimicrobial potential, our interest was also to see whether they were effective against biofilm formation. The antibiofilm efficacy of compounds **5a–f** was evaluated against Gram-positive (*Staphylococcus aureus, Enterococcus faecalis*) and Gram-negative bacteria (*Escherichia coli, Pseudomonas aeruginosa*) and several *Candida* species (*C. albicans*, *C. kefyr*, *C. krusei*, *C. parapsylosis, C. tropicalis*). The activity was compared to that of gentamycin, amphotericin B and the parent compound PQ (Table 2).

**Table 2.** Sensitivity of microbial strains to PQ-ureidoamides expressed as minimum biofilm eradication concentration (MBEC).

	MBEC (µg/ml)								
Microorganism	PQ	**5a**	**5b**	**5c**	**5d**	**5e**	**5f**	Gen	Amph
*Staphylococcus aureus* ATCC 25213	>100	25	>100	>100	25	>100	>100	12.5	na
*Staphylococcus aureus* ATCC 6538	50	12.5	>100	>100	25	>100	>100	12.5	na
*Enterococcus faecalis* ATCC 29212	>100	>100	>100	>100	>100	>100	>100	50	na
*Escherichia coli* ATCC 8739	25	6.25	12.5	6.25	25	50	6.25	25	na
*Escherichia coli* ATCC 10536	25	6.25	12.5	6.25	25	50	12.5	25	na
*Pseudomonas aeruginosa* ATCC 27853	50	25	>100	25	50	>100	>100	50	na
*Pseudomonas aeruginosa* ATCC 9027	70	60	>100	50	50	>100	>100	50	na
*Candida albicans* ATCC 10231	>100	>100	50	50	50	25	25	na	25
*Candida albicans* ATCC 90028	>100	25	50	25	25	25	25	na	25
*Candida kefyr* ATCC 2512	50	>100	>100	>100	25	6.25	6.25	na	25
*Candida krusei* ATCC 14243	>100	>100	>100	>100	>100	>100	>100	na	50
*Candida parapsylosis* ATCC 22019	50	>100	>100	>100	>100	>100	>100	50	na
*Candida tropicalis* ATCC 750	>100	>100	>100	>100	>100	>100	>100	na	50

Amph: amphotericin B; Gen: gentamycin; na: no activity; PQ: primaquine.

As shown in Table 2, antibiofilm potency of all compounds against the biofilm formed by *E. coli* (both strains) was prominent, with lower MBEC than the MBECs of gentamycin and PQ (25 µg/ml), especially for **5a** and **5c**. These two compounds were also very potent in eradicating the biofilm formed by *P. aeruginosa*. Compound **5a** had very low MBEC (at the level of gentamycin) for *S. aureus*. In contrast, we found that none of the tested compounds was effective against biofilm formation by *C. parapsylosis*, *E. faecalis, C. tropicalis* and *C. krusei*. As already mentioned, compounds **5a–f** had high efficiency against biofilm formation of Gram-negative bacteria *E. coli* (both tested strains). This finding is unexpected since Gram-negative bacteria have a more complex cell envelope, which is a relatively impermeable structure formed of an inner cytoplasmic membrane separated by a peptidoglycan layer from an outer envelope. One of the resistance mechanisms that differ Gram-negative from Gram-positive bacteria is the membrane^24^. Since the exact mechanism underlying PQ activity is unclear, one of the suggested possibilities of such activity is the formation of reactive oxidative species^25^, which could be the mechanism also involved in both antibacterial action and inhibition of biofilm formation. Tested PQ-derivatives, like the parent compound, might bind to specific sites within bacterial membranes and cause changes in membrane proteins, modulate their conformational status, and/or modify their lipid structure^26^. Presumably, this action is not specific but may play an important role in the mechanism of antibacterial action. To extend this, it would be of interest to address drug–membrane interactions at different levels so as to approach molecular events that might underlie antibacterial action of PQ derivatives. In addition, it is known that PQ interferes with DNA synthesis^26^. If this also occurs when we treat bacteria with PQ-derivatives, it could be possible that they depress the formation of an extracellular matrix that holds bacterial cells together in the biofilm, since the matrix is composed, among others, of protein and DNA^27^.

All compounds reduced biofilm formation of the yeast *Candida albicans* with MBEC values similar to or even lower than the standard antimycotic drug amphotericin B. It could be possible that PQ-ureidoamides, similar to the parent compound PQ, target Fe-S cluster proteins and affect yeast respiratory growth^28^. Compounds **5e** and **5f** showed higher antibiofilm potential against yeast, especially against biofilm formation of *C. kefyr* with a rather low MBEC (6.25 µg/ml). This non-albicans *Candida* strain is one of the species identified as a common cause of candidiasis as a nosocomial infection^29^. These infections are often connected with frequent use of broad-spectrum antibiotics and different medical devices (central venous catheters, urinary catheters, prosthetic devices), and the finding of an efficient antibiofilm agent is of importance in this aspect as well. Since PQ induces morphological changes in membranes of *P. fallax* and inhibition of functional transport vesicle formation in the Golgi apparatus^30^, one can speculate that a similar interaction may occur with the tested compounds. The study showed non-specific interactions of PQ (in the charged form) with the polar region of the phospholipids^26^. Thus, phospholipid–drug interactions may be the mechanism by which the tested compounds affect biofilm formation as well. It has been proposed that PQ-induced local changes of the lipid layer of the membrane may influence the protein function and start a signalling cascade that may lead to other biological events influencing the steady-state of a cell, in this case a bacterial or yeast cell^31^.

Free radicals play an important role in the inflammatory process and several diseases, as well as in potential damage of cellular compounds such as DNA, proteins and lipids. Consequently, compounds with antioxidative properties could be expected to offer protection and therapeutic treatment. LOX inhibitors are potential agents for the treatment of inflammatory and allergic diseases, certain types of cancer, and cardiovascular diseases^32^. Thus, to evaluate the antioxidative potential of PQ-derivatives **5a–f**, we have used three different antioxidant assays: (a) the interaction with the stable free radical DPPH, (b) the interaction with the water-soluble azo compound AAPH and (c) the inhibition of soybean lipoxygenase *in vitro*. DPPH interaction of the tested compounds was examined at a 100 µM concentration after 20 and 60 min. As shown in Table 3, DPPH-reducing ability of all tested compounds was very low, possibly due to stereochemical reasons.

**Table 3.** DPPH-reducing ability, IC_50_ values of soybean lipoxygenase (LOX) inhibition *in vitro* and anti-lipid peroxidation activity (LP) of compounds **5a–f**.

	DPPH-reducing ability^a^ (%)			
Compd.	20 min	60 min	LOX inhibition (IC_50_ μM) ± SD	LP inhibition^a^ (%) ± SD
**5a**	6^b^	5^b^	40.0 ± 1.0^a^^,^^c^	15 ± 0.7
**5b**	7^b^	5^b^	69.0 ± 2.0	92 ± 3.2
**5c**	2^b^	2^b^	40.0 ± 1.5	99 ± 2.8
**5d**	1^b^	2^b^	41.5 ± 3.1	90 ± 4.5
**5e**	1^b^	1^b^	56.0 ± 2.3	80 ± 2.7
**5f**	2^b^	2^b^	67.5 ± 1.1	90 ± 3.6
NDGA	83.0 ± 1.0	91.0 ± 1.7	0.45 ± 0.01	nt
Trolox	nt	nt	Nt	96 ± 2.0

^a^Concentration of the tested compounds: 100 μΜ.

^b^non-significant.

^c^per cent.

Nt: not tested; SD: standard deviation.

In the AAPH assay, the highly reactive alkylperoxyl radicals are intercepted mainly through a hydrogen atom transfer (HAT) from the antioxidant. Therefore, particularly effective HAT agents are compounds with a high hydrogen atom donating ability, that is, compounds with low heteroatom-H bond dissociation energies and/or compounds from which hydrogen abstraction leads to sterically hindered radicals as well as compounds from which abstraction of hydrogen leads to C-centered radicals stabilised by resonance. LOX inhibition experiments showed that all PQ-ureidoamides, with the exception of compound **5a**, were active, with IC_50_ values ranging from 40 to 69 µΜ. Again, derivative **5c** was the most potent inhibitor (IC_50_ = 40 µM), followed by **5d** (IC_50_ was slightly higher, e.g. 41.5 µM) and **5e** (IC_50_ = 56 µM). The results showed that the presence of a chloro substituent was beneficial to activity, while position of the chlorine atom had no influence. Compounds **5a** and **5b** without a chloro substituent showed lower activity. However, the presence of the second Cl atom (**5e**) and a combination of chloro and methoxy substituents (**5f**) diminished the activity. Although these results are preliminary, they suggest a possible anti-inflammatory effect related to an antioxidative ability. More studies are needed to establish the anti-inflammatory activity and their mechanism of action.

In further study, the potential of compounds **5a–f** to inhibit lipid peroxidation (LP) was evaluated as well. The results are given in Table 3. All derivatives inhibited lipid peroxidation significantly (80–99%), except for compound **5a** (derivative with an aliphatic chain in the amino acid region). Phenylglycine derivative **5c** with a chlorobenzhydryl substituent was the most potent (99%). Our results indicate that LOX inhibition was accompanied and correlated with LP inhibition.

PQ-ureidoamides **5a–f** with potential antibacterial application could be intended for injectable use and thus *in vitro* haemolytic study was performed to evaluate haemoglobin release as an indicator of red blood cell lysis. Such studies are needed to address possible aspects of compound toxicity. Haemolytic activity of compounds **5a–f** was evaluated using the fresh human erythrocyte assay, following the procedure described in experimental section (Table 4).

**Table 4.** Haemolytic activity of PQ-ureidoamides **5a–f**.

	Haemolysis (%)^a^							
Conc. (μg/ml)	**5a**^c^^,^^d^	**5b**^c^^,^^d^	**5c**^c^^,^^d^	**5d**^c^^,^^d^	**5e**^c^^,^^d^	**5f**^c^^,^^d^	PQ^c^^,^^d^	DMSO (1.5%)
200	4.24 ± 0.07^b^	4.04 ± 0.08^b^	4.89 ± 0.13^b^	3.07 ± 0.05^b^	3.70 ± 0.08^b^	4.03 ± 0.09^b^	3.12 ± 0.07	3.23 ± 0.06
100	4.03 ± 0.03^b^	3.94 ± 0.05^b^	4.26 ± 0.07^b^	2.94 ± 0.04^b^	3.23 ± 0.05^b^	3.66 ± 0.05^b^	2.53 ± 0.04	
50	3.14 ± 0.04^b^	3.13 ± 0.05^b^	3.06 ± 0.05^b^	2.56 ± 0.05^b^	2.78 ± 0.04^b^	3.32 ± 0.05^b^	2.08 ± 0.05	

^a^Mean ± SD of three measurements.

^b^Not significantly different from primaquine.

^c^Not significantly different from solvent.

^d^No significant difference between concentration tested (using one-way ANOVA with Dunnet's post-test).

Haemolytic activity tests showed no statistical differences between the tested PQ-derivatives and the parent compound PQ. The solvent DMSO (1.5%, *v/v*) did not cause significant haemolytic activity either. Overall, the tested compounds expressed no significant haemolytic activities in concentrations up to 200 µg/ml after 1 h of exposure to a 4% suspension of fresh human erythrocytes under aerobic conditions.

## Conclusions

The occurrence of drug-resistant strains of bacteria is rising, and therefore, new approaches to treat bacterial infections are emerging, particularly for Gram-negative bacteria. Our findings suggest that the PQ-ureidoamides tested in this study may offer a new option for the treatment of biofilm-associated infections. They prevent bacterial adhesion to the surface and diminish the formation of a biofilm on abiotic surfaces at the concentration level of referent antimicrobial drugs or even at lower concentrations that are considered relevant for possible future use *in vivo*. Compounds **5a–f** had high efficiency against biofilm formation of Gram-negative bacteria *E. coli* (MBEC from 6.25 to 50 µg/ml, four compounds had lower MBECs than gentamycin) and yeast *C. albicans* with MBEC values similar to or higher than the standard antimycotic drug amphotericin B. Compounds **5e** and **5f** showed higher antibiofilm potential against yeast, especially against biofilm formation of *C. kefyr* with a rather low MBEC (6.25 µg/ml). The favourable non-toxic profile of the PQ-ureidoamides tested on a series of human cancer cell lines and rat skeletal myoblasts^14^ is in line with the guidelines for novel antibiofilm drugs. However, further studies are needed to clarify the mechanism of the antibiofilm effect observed. Some of our compounds kill bacteria at their planktonic stage as well: leucine derivative **5a** and *p*-chlorophenylglycine PQ-ureidoamide **5d** showed significant antibacterial activity against three *S. aureus* strains (MIC = 6.5 µg/ml), while compounds **5c** and **5d** showed weak antibacterial activity against *E. coli* and *P. aeruginosa*. Finally, compounds **5b–f** significantly expressed anti-lipid peroxidation activity, whereas phenylglycine derivative **5c** with a chlorobenzhydryl substituent exhibited antioxidant and anti-LOX profile.

## Disclose statement

The research was conducted in the absence of any commercial or financial relationships that could be construed as a potential conflict of interest.

## Supplementary Material

IENZ_1423067_Supplementary_Materials.pdf
