# Supplementary material for: Insights into biological activity of ureidoamides with primaquine and amino acid moieties
Source: J Enzyme Inhib Med Chem. 2018 Jan 24;33(1):376–82. doi: 10.1080/14756366.2017.1423067 (PMC6021035; doi:10.1080/14756366.2017.1423067)
Supplement: IENZ_1423067_Supplementary_Materials.pdf [file IENZ_A_1423067_SM2283.pdf]

Supplementary material

1. Synthesis of compounds 5a-f and their precursors

*Btc-amino acids (**2a-c**): general procedure.* To a suspension of 20 mmol of amino acid in dry dioxane (40 ml) a solution of 1.81 g (10 mmol) BtcCl (**1**) in dry dioxane (10 ml) was added dropwise. The reaction mixture was stirred overnight at room temperature. Amino acid hydrochloride was filtered off, washed with dioxane and the mother liquor was evaporated under reduced pressure. The crude products were triturated several times with hot toluene. 2-[(1*H*-1,2,3-benzotriazole-1-carbonyl)amino]-4-methylpentanoic acid (Btc-L-leucine, **2a**) and 2-[(1*H*-1,2,3-benzotriazole-1-carbonyl)amino]-2-phenylacetic acid (Btc-D-phenylglycine, **2b**) were described in our previous paper[1], while 2-[(1*H*-1,2,3-benzotriazole-1-carbonyl)amino]-2-(4-chlorophenyl)acetic acid (Btc-DL-*p*-chlorophenylglycine, **2c**) is a new compound.

*2-[(1H-1,2,3-benzotriazole-1-carbonyl)amino]-2-(4-chlorophenyl)acetic acid (Btc-DL-p-chlorophenylglycine (**2c**).* From the reaction of 3.712 g *p*-chlorophenylglycine and 1.81 g chloride **1**, 3.274 g (99%) of **2c** was obtained; mp 127 °C (decomp.); IR (KBr): ν_{max} 3360, 2924, 2540, 1750, 1712, 1494, 1450, 1380, 1290, 1184, 1136, 1064, 1018, 976, 932, 822, 786, 754, 732, 680, 662, 642, 624, 600, 558, 538, 516, 466 cm^{-1}; ^{1}H NMR (DMSO-d_6) δ 9.63-9.62 (d, 1H, 10', *J* = 7.21), 8.24-7.15 (m, 9H, 3-6, 1', 5', 6', 8', 9'), 5.71-5.69 (d, 1H, 2', *J* = 7.20); ^{13}C NMR (DMSO-d_6) δ 170.66 (3'), 148.50 (1), 145.48 (7), 135.76 (4'), 132.81 (7'), 131.25 (2), 130.22 (4), 130.14, 128.41 (5', 6', 8', 9'), 125.76 (5), 119.94 (6), 113.38 (3), 56.69 (2'); MS/MS *m/z* 120.1 $[BtH+1]^+$, 353.2 $[M+Na]^+$.

*Btc-amino acid chlorides (**3a-c**): general procedure*

A solution of 4 mmol Btc-amino acid in 20 ml thionyl chloride was stirred overnight at room temperature. Thionyl chloride was evaporated under reduced pressure and the residue was

dissolved in dry toluene and evaporated again (several times). Crude products **3a-c** were used immediately in further reactions.

*Btc-amino acid amides (**4a-f**): general procedure*

A solution of 3.4 mmol corresponding amine or hydrazone and 4 mmol TEA in dry toluene (20 ml) was added dropwise to a solution of 4 mmol chloride **3** in dry toluene (30 ml). The reaction mixture was stirred 30 min at room temperature. TEA × HCl was filtered off and mother liquor was evaporated. The residue was dissolved in ethyl acetate/1% HCl mixture (1:1). The organic layer was extracted three times with HCl solution (w = 1%), washed two times with water, dried over anhydrous sodium sulfate, filtered and evaporated under reduced pressure.

*2-[(1H-1,2,3-benzotriazole-1-carbonyl)amino]-N-(diphenylmethyl)-4-methylpentanamide (**4a**).* From the reaction of 0.623 g diphenylmethanamine, 1.179 g chloride **3a** and 0.404 g TEA, and after trituration with ether, 1.081 g (72%) of **4a** was obtained; mp 155–158 °C; IR (KBr): ν_{max} 3328, 3261, 3068, 2958, 2929, 1716, 1650, 1557, 1522, 1450, 1378, 1287, 1230, 1152, 1079, 1050, 1013, 925, 855, 836, 753, 699, 633, 567, 521, 462 cm^{-1}; ^{1}H NMR (DMSO-d_6) δ 9.12-9.09 (2d, 2H, 1', 1'', J = 3.95), 8.23-7.24 (m, 14H, 3-6, 4''-8'', 10''-14''), 6.18-6.16 (d, 1H, 2'', J = 8.37), 4.71-4.65 (m, 1H, 2'), 1.94-1.88 (m, 1H, 4'), 1.73-1.62 (m, 2H, 5'), 0.95-0.89 (d, 6H, 6', 7', J = 6.48); ^{13}C NMR (DMSO-d_6) δ 170.58 (3'), 148.84 (1), 145.47 (7), 142.19 (3'', 9''), 131.32 (2), 130.06 (4), 128.40-127.05 (4''-8'', 10''-14''), 125.65 (5), 119.84 (6), 113.54 (3), 56.08 (2'), 52.97 (2''), 38.89 (4'), 24.46 (5'), 23.08, 21.38 (6', 7'); MS/MS m/z 120.1 [BtH+1]$^{+}$[31].

*2-[(1H-1,2,3-benzotriazole-1-carbonyl)amino]-N-(diphenylmethyl)-N-methyl-2-phenylacetamide (**4b**).* From the reaction of 0.671 g *N*-methyl-1,1-diphenylmethanamine, 1.259 g chloride **3b** and 0.404 g TEA, and after purification by column chromatography

(mobile phase cyclohexane/ethyl acetate/methanol 30:10:5), 0.857 g (53%) of **4b** was obtained; mp 82–83.5 °C; IR (KBr): ν_{max} 3377, 3032, 2939, 1735, 1649, 1490, 1405, 1294, 1231, 1081, 1033, 932, 867, 813, 753, 702, 611, 569, 519 cm^{-1}; ^{1}H NMR (DMSO-d_6) δ 9.16-9.14 (d, 1H, 1', J = 5.97), 8.22-6.93 (m, 19H, 5'-9', 3-6, 4"-8", 10"-14"), 6.53 (s, 1H, 2"), 6.16-6.14 (d, 1H, 2', J = 6.23), 2.67 (s, 3H, 1"); ^{13}C NMR (DMSO-d_6) δ 169.24 (3'), 147.40 (1), 145.50 (7), 138.47, 138.24 (3", 9"), 135.92 (4'), 131.08 (2), 130.26 (4), 128.80-127.51 (5'-9', 4"-8", 10"-14"), 125.71 (5), 119.97 (6), 113.28 (3), 61.00 (2"), 55.58 (2'), 31.69 (1"); MS/MS *m/z* 117.9 [BtH–1]$^-$.

*2-[(1H-1,2,3-benzotriazole-1-carbonyl)amino]-N-[(4-chlorophenyl)(phenyl)methyl]-2-phenylacetamide (**4c**).* From the reaction of 0.740 g (4-chlorophenyl)(phenyl)methanamine, 1.259 g chloride **3b** and 0.404 g TEA, and after trituration with ether/petroleum ether and purification of mother liquid by column chromatography (mobile phase cyclohexane/ethyl acetate/methanol 30:10:5), 1.400 g (83%) of **4c** was obtained; mp 116–118.5 °C; IR (KBr): ν_{max} 3286, 3062, 3032, 1748, 1660, 1502, 1499, 1448, 1380, 1232, 1182, 1126, 1094, 1036, 934, 852, 812, 752, 698, 612, 572, 554, 518, 496 cm^{-1}; ^{1}H NMR (DMSO-d_6) δ 9.41-9.39 (t, 1H, 1", J = 7.41), 9.03-9.01 (d, 1H, 1', J = 7.14), 8.23-7.08 (m, 18H, 5'-9', 3-6, 4", 5", 7", 8", 10"-14"), 6.19-6.17 (d, 1H, 2", J = 8.10), 5.84-5.81 (t, 1H, 2', J = 6.46); ^{13}C NMR (DMSO-d_6) δ 172.20 (3'), 148.06 (1), 145.50 (7), 141.21, 140.76 (3", 9"), 135.60 (4'), 132.20 (6"), 131.15 (2), 130.21 (4), 129.40-126.72 (5'-9', 4", 5", 7", 8", 10"-14"), 125.69 (5), 119.91 (6), 113.33 (3), 59.56 (2'), 56.54 (2"); MS/MS *m/z* 120.1 [BtH+1]$^+$, 496.2 [M+1]$^+$.

*2-[(1H-1,2,3-benzotriazole-1-carbonyl)amino]-2-(4-chlorophenyl)-N-(diphenylmethyl)acetamide (**4d**).* From the reaction of 0.623 g diphenylmethanamine, 1.397 g chloride **3c** and 0.404 g TEA, and after trituration several times with ether/petroleum ether, 0.640 g (38%) of **4d** was obtained; mp 164.5–166.5 °C; IR (KBr): ν_{max} 3388, 3276, 3062,

1732, 1682, 1648, 1492, 1448, 1380, 1288, 1218, 1094, 1024, 930, 818, 750, 702, 648, 590, 566, 540 cm^{-1}; ^{1}H NMR (DMSO-d_6) δ 9.41-9.40 (d, 1H, 1', J = 8.23), 9.10-9.09 (d, 1H, 1'', J = 7.29), 8.23-7.08 (m, 18H, 3-6, 5', 6', 8', 9', 4''-8'', 10''-14''), 6.17-6.15 (d, 1H, 2'', J = 8.20), 5.84-5.83 (d, 1H, 2', J = 7.23); ^{13}C NMR (DMSO-d_6) δ 167.71 (3'), 148.10 (1), 145.47 (7), 141.62, 141.60 (3'', 9''), 136.63 (4'), 132.79 (7'), 131.15 (2), 130.18 (4), 129.27-126.85 (5', 6', 8', 9', 4''-8'', 10''-14''), 125.69 (5), 119.90 (6), 113.31 (3), 56.55 (2'), 56.34 (2''); MS/MS *m/z* 120.1 $[BtH+1]^+$, 496.2 $[M+1]^+$.

2-[(1H-1,2,3-benzotriazole-1-carbonyl)amino]-2-(4-chlorophenyl)-N-[(4-chlorophenyl)(phenyl)methyl]acetamide ***(4e)***. From the reaction of 0.740 g (4-chlorophenyl)(phenyl)methanamine, 1.397 g chloride **3c** and 0.404 g TEA, and after purification by column chromatography (mobile phase cyclohexane/ethyl acetate/methanol 30:10:5) and trituration with ether/petroleum ether, 0.307 g (17%) of **4e** was obtained; mp 158–159.5 ^{o}C; IR (KBr): ν_{max} 3394, 3279, 3062, 1730, 1651, 1492, 1450, 1410, 1381, 1321, 1290, 1219, 1184, 1150, 1093, 1017, 930, 828, 753, 702, 623, 563, 537 cm^{-1}; MS/MS *m/z* 120.2 $[BtH+1]^+$. The structure of product **4e** was confirmed indirectly, by chemical derivatization to compound **5e**.

N-[(4-chlorophenyl)({N'-[(4-methoxyphenyl)(phenyl)methylidene]hydrazinecarbonyl})methyl]-1H-1,2,3-benzotriazole-1-carboxamide ***(4f)***. From the reaction of 0.769 g 4-methoxybenzophenone hydrazone, 1.397 g chloride **3c** and 0.404 g TEA, and after purification by column chromatography (mobile phase cyclohexane/ethyl acetate/methanol 30:10:5) and trituration with ether, 1.136 g (62%) of **4f** was obtained; IR (KBr): ν_{max} 3373, 3307, 3181, 3076, 3049, 2923, 2829, 1733, 1676, 1604, 1506, 1489, 1443, 1377, 1305, 1289, 1256, 1179, 1157, 1108, 1089, 1031, 960, 935, 836, 806,

779, 749, 696, 653, 628, 603, 570, 532 cm^{-1}; MS/MS *m/z* 120.1 $[BtH+1]^+$, 539.1 $[M+1]^+$. The structure of product **4f** was confirmed indirectly, by chemical derivatization to compound **5f**.

*PQ-ureidoamides (**5a-f**): general procedure*

Method A: NaOH solution (*w* = 5%) was added to a solution of 1 mmol PQ diphosphate in water until pH 9-10 was reached, and PQ base was extracted four times with dichloromethane and washed with water until pH 7. The organic layer was dried over anhydrous sodium sulfate, filtered and evaporated under reduced pressure. PQ base and 0.5 mmol Btc-amino acid amide **4** were mixed in dioxane and stirred overnight at room temperature, light protected. The solvent was evaporated under reduced pressure.

Method B: A suspension of 0.4 mmol PQ diphosphate, 0.4 mmol Btc-amino acid amide **4b** and 0.8 mmol TEA in dichloromethane (3 mL) was heated at 70 °C in microwave reactor for 45 min. The solvent was removed under reduced pressure. The residue was dissolved in ethyl acetate/5% NaOH mixture (1:1). The organic layer was extracted with 5% NaOH three times, washed with water two times, dried over anhydrous sodium sulfate, filtered and evaporated.

*N-(diphenylmethyl)-2-[({4-[(6-methoxyquinolin-8-yl)amino]pentyl}carbamoyl)amino]-4-methylpentanamide (**5a**).* Method A, from the reaction of 0.221 g Btc-amino acid amide **4a** and 0.259 g PQ, and after purification by column chromatography (mobile phase cyclohexane/ethyl acetate/methanol 30:10:5) and trituration with ether, 0.253 g (87%) of **5a** was obtained; mp 184.5–185.5 °C; IR (KBr): ν_{max} 3316, 2958, 1655, 1624, 1569, 1519, 1493, 1454, 1422, 1388, 1336, 1290, 1201, 1167, 1155, 1052, 1028, 824, 791, 746, 697 cm^{-1}; ^1H NMR (DMSO-d_6) δ 8.89-8.87 (d, 1H, 1'', *J* = 8.53), 8.52 (s, 1H, 11), 8.08-8.06 (d, 1H, 13, *J* = 8.25), 7.43-7.20 (m, 11H, 12, 4''-8'', 10''-14''), 6.46 (s, 1H, 17), 6.25 (s, 1H, 15), 6.12-5.97 (m, 4H, 2, 8, 1', 2''), 4.33-4.32 (q, 1H, 2', *J* = 8.05, 14.91), 3.81 (s, 3H, 18), 3.60 (m, 1H, 6), 3.00 (m, 2H, 3), 1.61-1.35 (m, 7H, 4, 5, 4', 5'), 1.18 (d, 3H, 7, *J* = 3.31), 0.85-0.82 (2d, 6H, 6', 7');

^{13}C NMR (DMSO-d_6) δ 172.46 (3'), 159.02 (16), 157.62 (1), 144.64 (9), 144.25 (11), 142.45, 142.40 (3'', 9''), 134.82 (13), 134.54 (10), 129.60 (14), 128.34-126.93 (4''-8'', 10''-14''), 122.12 (12), 96.12 (17), 91.61 (15), 55.73 (2'), 55.00 (18), 51.44 (2''), 47.05 (6), 42.22 (4'), 39.02 (3), 33.53 (5), 26.93 (4), 24.27 (5'), 23.03, 22.01 (6', 7'), 20.24 (7); MS/MS *m/z* 582.2 [M+1]$^+$. Anal. Calcd. for ($C_{35}H_{43}N_5O_3$): C, 72.26; H, 7.45; N, 12.04. Found: C, 72.50; H, 7.47; N, 12.09.

*N-(diphenylmethyl)-2-[({4-[(6-methoxyquinolin-8-yl)amino]pentyl}carbamoyl)amino]-N-methyl-2-phenylacetamide (**5b**).* Method B, from the reaction of 0.190 g Btc-amino acid amide **4b**, 0.182 g PQ diphosphate and 0.081 g TEA, and after purification by column chromatography (mobile phase cyclohexane/ethyl acetate/methanol 30:10:5) and trituration with ether, 0.084 g (34%) of **5b** was obtained; mp 146.5–148.5 °C; IR (KBr): ν_{max} 3335, 3060, 3030, 2962, 2933, 2863, 1622, 1563, 1521, 1495, 1479, 1457, 1406, 1388, 1337, 1308, 1221, 1201, 1163, 1116, 1082, 1052, 1031, 1003, 971, 921, 869, 823, 791, 769, 735, 720, 701, 678, 624, 608, 566, 467 cm^{-1}; ^{1}H NMR (DMSO-d_6) δ 8.54-8.53 (d, 1H, 11, *J* = 3.06), 8.09-8.07 (d, 1H, 13, *J* = 7.94), 7.42-6.89 (m, 16H, 12, 5'-9', 4''-8'', 10''-14''), 6.67-6.46 (m, 3H, 17, 1', 2''), 6.25-6.09 (2s, 2H, 2, 15), 6.12-6.09 (d, 1H, 8, *J* = 8.50), 5.77-5.75 (d, 1H, 2', *J* = 8.11), 3.82 (s, 3H, 18), 3.61 (m, 1H, 6), 3.01 (m, 2H, 3), 2.65 (s, 3H, 1''), 1.61-1.48 (4, 5), 1.19-1.18 (d, 3H, 7, *J* = 5.83); ^{13}C NMR (DMSO-d_6) δ 171.34 (3'), 159.00 (16), 157.07 (1), 144.63 (9), 144.24 (11), 138.81, 138.62 (4, 3'', 9''), 134.80 (13), 134.51 (10), 129.58 (14), 128.66-127.36 (5'-9', 4''-8'', 10''-14''), 122.10 (12), 96.11 (17), 91.62 (15), 60.33 (2''), 54.96 (18), 54.26 (2'), 47.00 (6), 39.02 (3), 33.52 (5), 31.69 (1''), 26.87 (4), 20.21 (7); MS/MS *m/z* 616.5 [M+1]$^+$. Anal. Calcd. for ($C_{38}H_{41}N_5O_3$): C, 74.12; H, 6.71; N, 11.37. Found: C, 74.39; H, 6.72; N, 11.33.

*N-[(4-chlorophenyl)(phenyl)methyl]-2-[({4-[(6-methoxyquinolin-8-yl)amino]pentyl}carbamoyl)amino]-2-phenylacetamide (***5c***).* Method A, from the reaction of 0.322 g (0.65 mmol) Btc-amino acid amide **4c** and 0.259 g PQ, and after purification by column chromatography (mobile phase cyclohexane/ethyl acetate/methanol 30:10:5) and trituration with ether, 0.351 g (85%) of **5c** was obtained; mp 129–131 °C; IR (KBr): ν_{max} 3317, 3255, 1621, 1571, 1547, 1518, 1489, 1453, 1422, 1386, 1218, 1157, 1090, 1051, 1029, 1014, 818, 790, 697 cm^{-1}; ^{1}H NMR (DMSO-d_6) δ 9.28-9.25 (dd, 1H, 1'', J = 5.74, 8.25), 8.54-8.53 (d, 1H, 11, J = 4.09), 8.09-8.06 (dd, 1H, 13, J = 1.41, 8.27), 7.44-7.09 (m, 15H, 12, 5'-9', 4'', 5'', 7'', 8'', 10''-14''), 6.69-6.65 (dd, 1H, 1', J = 3.84, 8.57), 6.48 (s, 1H, 17), 6.26-6.24 (2s, 2H, 2, 15), 6.12-6.07 (m, 2H, 8, 2''), 5.53-5.50 (dd, 1H, 2', J = 5.50, 8.37), 3.82 (s, 3H, 18), 3.61 (m, 1H, 6), 3.01 (m, 2H, 3), 1.61-1.44 (m, 4H, 4, 5), 1.19-1.18 (d, 3H, 7, J = 6.25); ^{13}C NMR (DMSO-d_6) δ 170.08 (3'), 159.01 (16), 157.09 (1), 144.64 (9), 144.26 (11), 141.66-141.09 (3'', 9''), 140.25 (4'), 134.82 (13), 134.53 (10), 131.75 (6''), 129.60 (14), 129.37-126.58 (5'-9', 4'', 5'', 7'', 8'', 10''–14''), 122.13 (12), 96.12 (17), 91.63 (15), 56.39 (2'), 55.26 (2''), 55.00 (18), 47.03 (6), 39.02 (3), 33.52 (5), 26.85 (4), 20.23 (7); MS/MS *m/z* 636.2 [M+1]$^{+}$; Anal. Calcd. for ($C_{37}H_{38}ClN_5O_3$): C, 69.85; H, 6.02; N, 11.01. Found: C, 69.73; H, 6.00; N, 10.99.

*2-(4-Chlorophenyl)-N-(diphenylmethyl)-2-[({4-[(6-methoxyquinolin-8-yl)amino]pentyl}carbamoyl)amino]acetamide (***5d***).* Method A, from the reaction of 0.230 g Btc-amino acid amide **4d** and 0.259 g PQ, and after purification by column chromatography (mobile phase cyclohexane/ethyl acetate/methanol 30:10:5) and trituration with ether, 0.114 g (36%) of **5d** was obtained; mp 180–183 °C; IR (KBr): ν_{max} 3321, 3254, 1621, 1572, 1547, 1519, 1491, 1455, 1422, 1388, 1348, 1218, 1197, 1159, 1091, 1051, 1030, 1013, 819, 790, 744, 693 cm^{-1}; ^{1}H NMR (DMSO-d_6) δ 9.27-9.25 (d, 1H, 1'', J = 8.53), 8.53-8.52 (d, 1H, 11, J = 3.74), 8.08-8.06 (dd, 1H, 13, J = 1.46, 8.24), 7.43-7.09 (m, 15H, 12, 5', 6', 8', 9', 4''-8'', 10''-14''), 6.70-6.69 (d, 1H, 1', J = 8.53), 6.47 (s, 1H, 17), 6.26-6.24 (2s, 2H, 2, 15), 6.11-6.10 (d,

1H, 8, *J* = 8.70), 6.06-6.05 (d, 1H, 2'', *J* = 8.49), 5.54-5.52 (dd, 1H, 2', *J* = 2.55, 8.52), 3.81 (s, 3H, 18), 3.62-3.60 (m, 1H, 6), 3.02-2.97 (m, 2H, 3), 1.64-1.41 (m, 4H, 4, 5), 1.19-1.18 (d, 3H, 7, *J* = 5.54); ^{13}C NMR (DMSO-d_6) δ 169.49 (3'), 158.98 (16), 156.92 (1), 144.61 (9), 144.21 (11), 142.01-141.86 (3'', 9''), 139.47 (4'), 134.77 (13), 134.49 (10), 131.81 (7'), 129.55 (14), 128.39-126.80 (5', 6', 8', 9', 4''-8'', 10''–14''), 122.06 (12), 96.06 (17), 91.62 (15), 56.90 (2'), 55.70 (2''), 54.95 (18), 47.00 (6), 39.02 (3), 33.47 (5), 26.78 (4), 20.18 (7); MS/MS *m/z* 636.4 $[M+1]^+$. Anal. Calcd. for ($C_{37}H_{38}ClN_5O_3$): C, 69.85; H, 6.02; N, 11.01. Found: C, 69.79; H, 6.01; N, 11.00.

*2-(4-Chlorophenyl)-N-[(4-chlorophenyl)(phenyl)methyl]-2-[({4-[(6-methoxyquinolin-8-yl)amino]pentyl}carbamoyl)amino]acetamide (**5e**).* Method A, from the reaction of 0.265 g Btc-amino acid amide **4e** and 0.259 g PQ, and after purification by column chromatography (mobile phase cyclohexane/ethyl acetate/methanol 30:10:5) and trituration with ether, 0.127 g (38%) of **5e** was obtained; mp 150–151.5 °C; IR (KBr): ν_{max} 3317, 3252, 1623, 1519, 1489, 1454, 1387, 1218, 1158, 1091, 1051, 1013, 819, 790, 757, 697 cm^{-1}; ^{1}H NMR (DMSO-d_6) δ 9.29-9.28 (d, 1H, 1'', *J* = 8.62), 8.53 (s, 1H, 11), 8.08-8.07 (d, 1H, 13, *J* = 7.19), 7.43-7.09 (m, 14H, 12, 5', 6', 8', 9', 4'', 5'', 7'', 8'', 10''-14''), 6.71-6.68 (dd, 1H, 1', *J* = 4.04, 8.40), 6.47 (s, 1H, 17), 6.26-6.24 (2s, 2H, 2, 15), 6.11-6.10 (d, 1H, 8, *J* = 8.66), 6.07-6.06 (d, 1H, 2'', *J* = 8.28), 5.53-5.50 (td, 1H, 2', *J* = 2.30, 8.23, 8.05), 3.81 (s, 3H, 18), 3.61-3.60 (m, 1H, 6), 3.00 (m, 2H, 3), 1.63-1.42 (m, 4H, 4, 5), 1.19-1.18 (d, 3H, 7, *J* = 6.22); ^{13}C NMR (DMSO-d_6) δ 169.59 (3'), 158.98 (16), 156.94 (1), 144.61 (9), 144.21 (11), 141.50-140.92 (3'', 9''), 139.32 (4'), 134.76 (13), 134.49 (10), 131.81, 131.56 (7', 6''), 129.55 (14), 129.27-126.84 (5', 6', 8', 9', 4'', 5'', 7'', 8'', 10''–14''), 122.06 (12), 96.08 (17), 91.62 (15), 55.77 (2'), 55.34 (2''), 54.95 (18), 47.00 (6), 39.02 (3), 33.47 (5), 26.76 (4), 20.18 (7); MS/MS *m/z* 670.4 $[M+1]^+$; Anal. Calcd. for ($C_{37}H_{37}Cl_2N_5O_3$): C, 66.27; H, 5.56; N, 10.44. Found: C, 66.40; H, 5.58; N, 10.46.

*3-[(4-Chlorophenyl)({N'-[(4-methoxyphenyl)(phenyl)methylidene]hydrazinecarbonyl})methyl]-1-{4-[(6-methoxyquinolin-8-yl)amino]pentyl}urea (**5f**)*. Method A, from the reaction of 0.269 g Btc-amino acid amide **4f** and 0.259 g PQ, and after purification by column chromatography (mobile phase dichloromethane/methanol 95:5 and cyclohexane/ethyl acetate 1:1) and trituration with ether, 0.068 g (20%) of oil product **5f** was obtained; IR (film): ν_{max} 3349, 2930, 1633, 1610, 1555, 1509, 1454, 1421, 1386, 1324, 1304, 1250, 1220, 1201, 1164, 1090, 1029, 959, 819, 790, 772, 697 cm^{-1}; ^{1}H NMR (DMSO-d_6) δ 10.28 (s, 1H, 1''), 8.54-8.53 (d, 1H, 11, J = 4.08), 8.09-8.06 (d, 1H, 13, J = 8.20), 7.58-6.92 (m, 14H, 12, 5', 6', 8', 9', 4'', 5'', 7'', 8'', 10''-14''), 6.74-6.71 (d, 1H, 1', J = 8.38), 6.48 (s, 1H, 17), 6.27-6.21 (m, 2H, 2, 15), 6.13-6.10 (d, 1H, 8, J = 8.71), 5.54-5.51 (d, 0.63H, 2', J = 7.95), 5.37-5.35 (d, 0.37H, 2', J = 7.81), 3.82 (s, 3H, 18), 3.86-3.84 (d, 1H, 16'', J = 4.06), 3.78-3.76 (d, 2H, 15'', J = 5.04), 3.61 (m, 1H, 6), 3.0 (m, 2H, 3), 1.65-1.48 (m, 4H, 4, 5), 1.21-1.19 (d, 3H, 7, J = 6.05); ^{13}C NMR (DMSO-d_6) δ 169.45 (3'), 167.10 (6''), 158.99 (16), 157.00 (1), 155.00 (2''), 144.62 (9), 144.24 (11), 138.90 (4'), 134.79 (13), 134.51 (10), 132.50 (9''), 131.98 (7'), 130.17-127.65 (5', 6', 8', 9', 4'', 8'', 10''-14''), 129.57 (14), 124.27 (3''), 122.09 (12), 114.60, 113.77 (5'', 7''), 96.09 (17), 91.62 (15), 55.21 (2'), 54.97 (18, 15''), 47.0 (6), 39.02 (3), 33.48 (5), 26.78 (4), 20.20 (7); MS/MS *m/z* 679.3 $[M+1]^+$; Anal. Calcd. for ($C_{38}H_{39}ClN_6O_4$): C, 67.20; H, 5.79; N, 12.37. Found: C, 67.39; H, 5.80; N, 12.40.

2. Spectra

2c

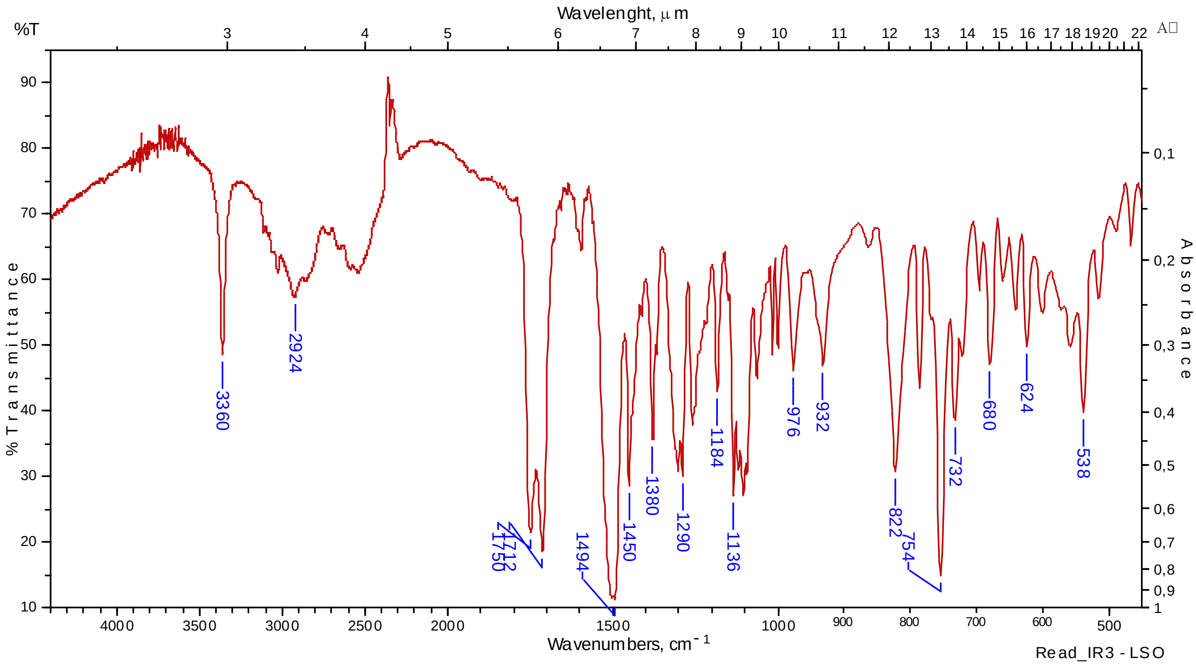

Wavelenght, µ m
%T
% Transmittance
Absorbance
Wavenumbers, cm⁻¹
Read_IR3 - LSO
3360
2924
1750
1712
1494
1450
1380
1290
1184
1136
976
932
822
754
732
680
624
538

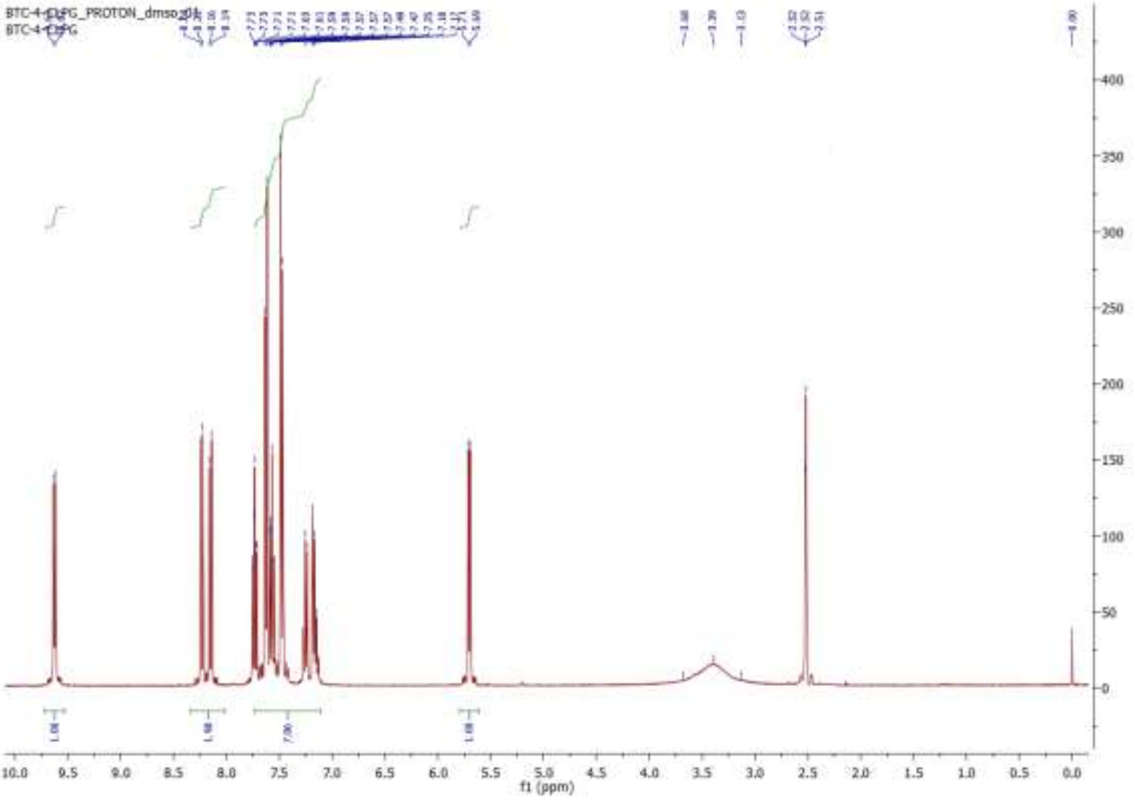

f1 (ppm)

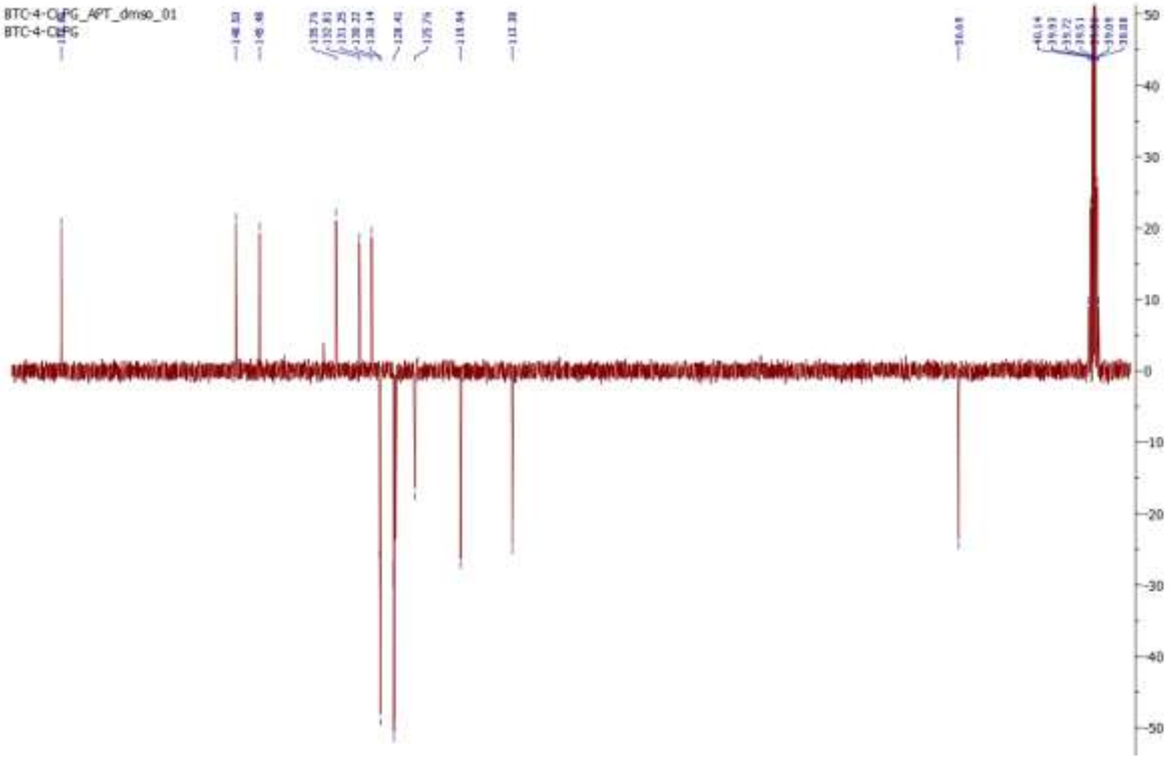
50
40
30
20
10
0
-10
-20
-30
-40
-50

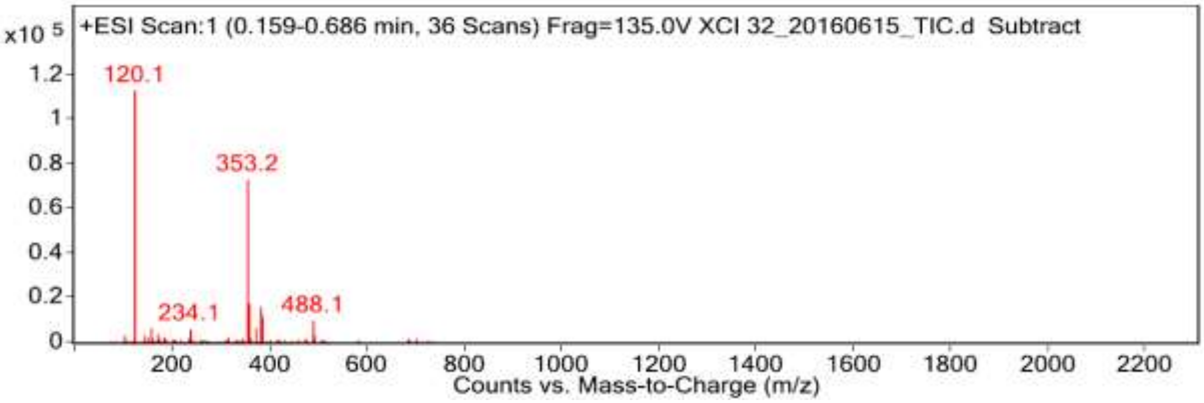
x10 5
+ESI Scan:1 (0.159-0.686 min, 36 Scans) Frag=135.0V XCI 32_20160615_TIC.d Subtract
1.2
1
0.8
0.6
0.4
0.2
0
120.1
353.2
234.1
488.1
200
400
600
800
1000
1200
1400
1600
1800
2000
2200
Counts vs. Mass-to-Charge (m/z)

4a

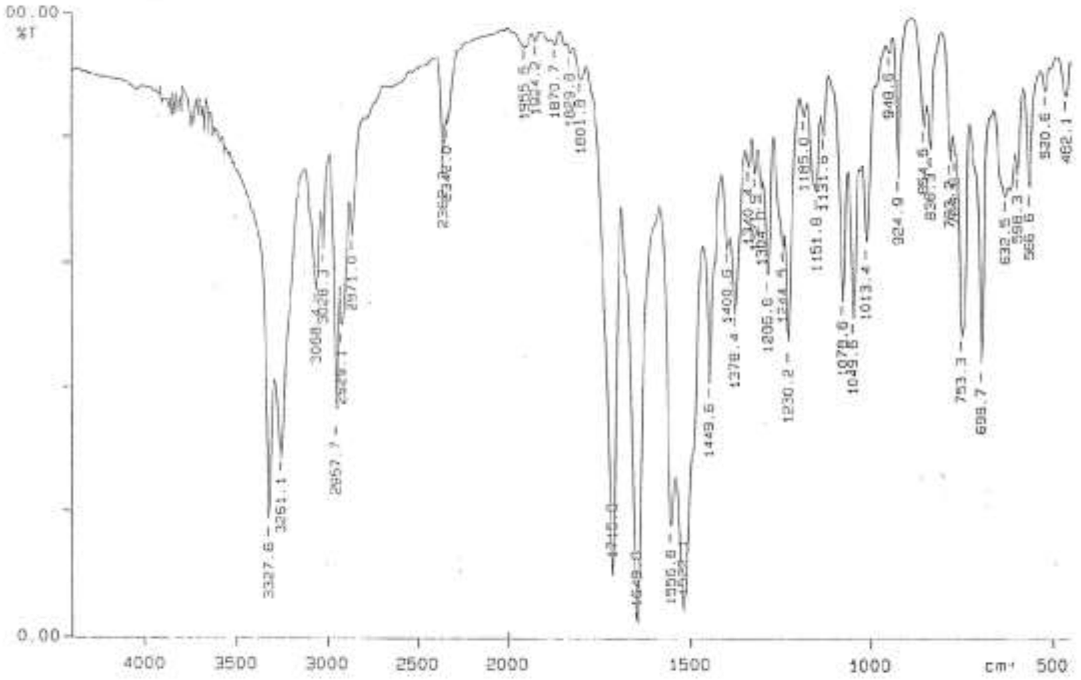

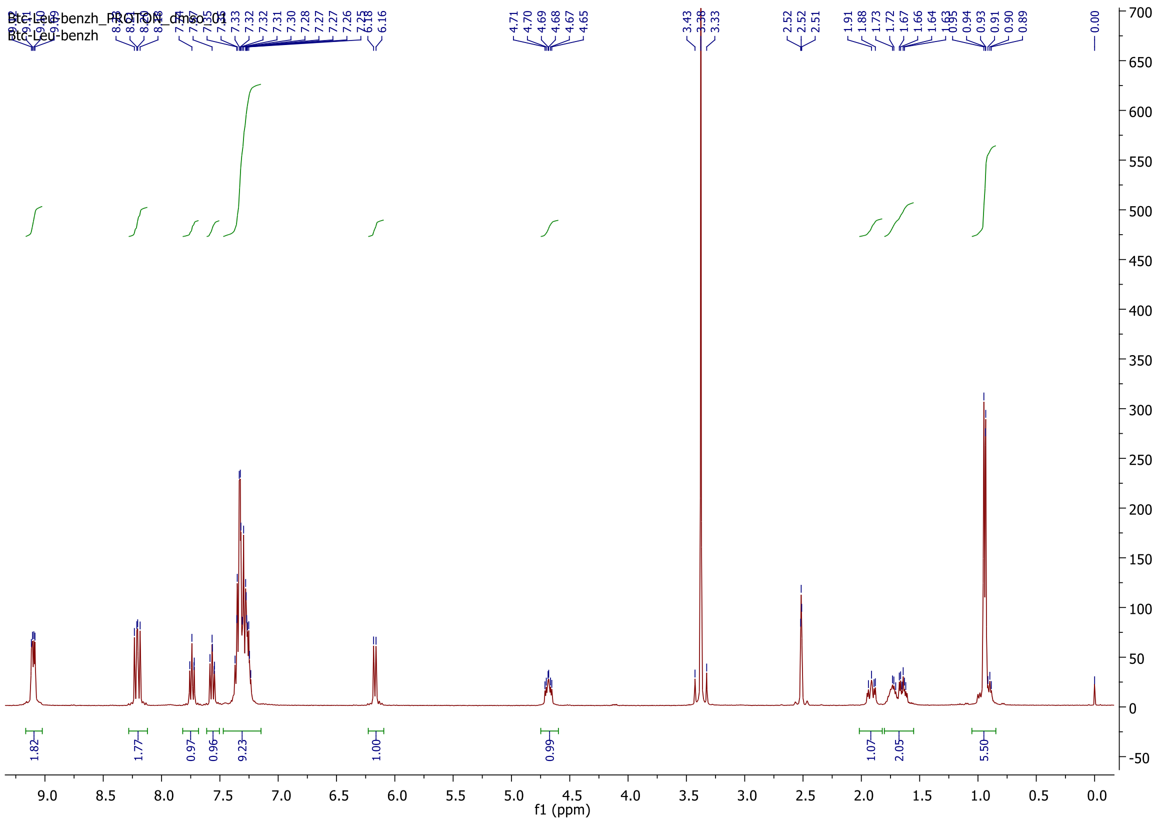

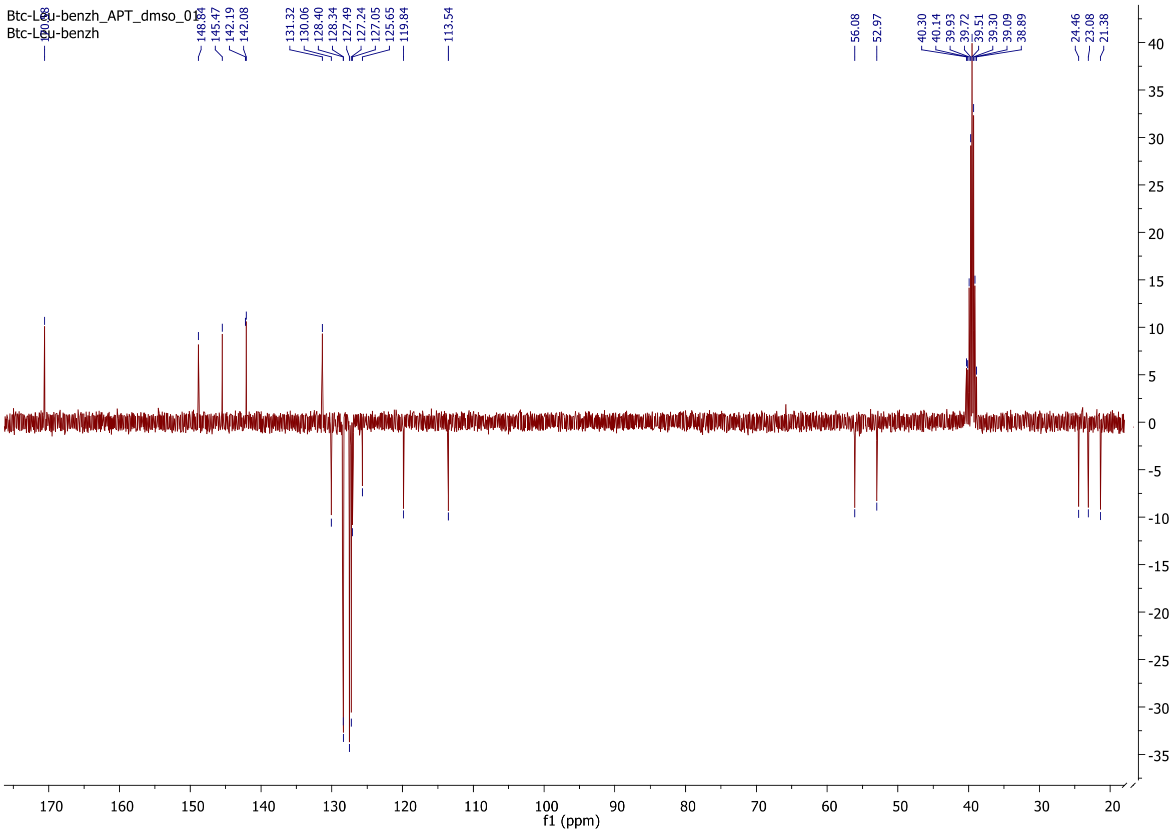
Btc-Leu-benzh_APT_dmso_01
Btc-Leu-benzh
148.84
145.47
142.19
142.08
131.32
130.06
128.40
128.34
127.49
127.24
127.05
125.65
119.84
113.54
56.08
52.97
40.30
40.14
39.93
39.72
39.51
39.30
39.09
38.89
24.46
23.08
21.38
f1 (ppm)

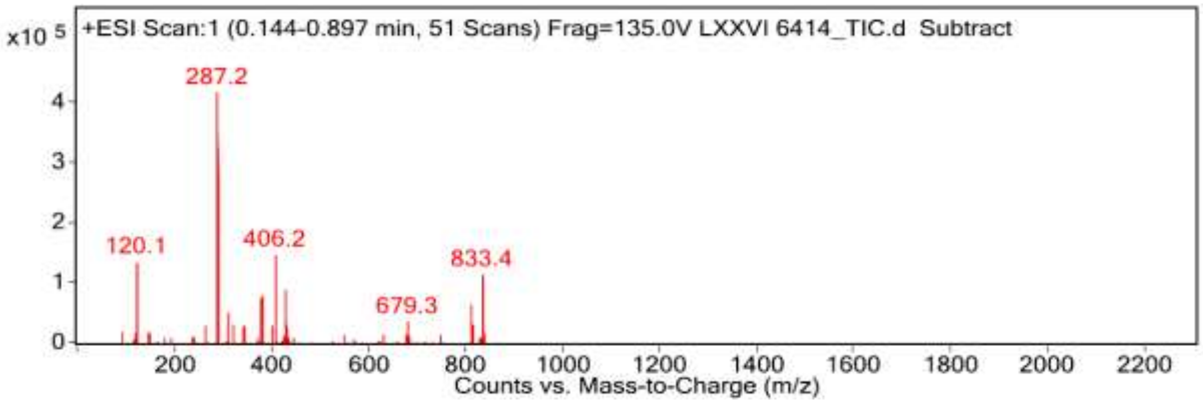
x10 5
+ESI Scan:1 (0.144-0.897 min, 51 Scans) Frag=135.0V LXXVI 6414_TIC.d Subtract
287.2
120.1
406.2
833.4
679.3
Counts vs. Mass-to-Charge (m/z)

4b

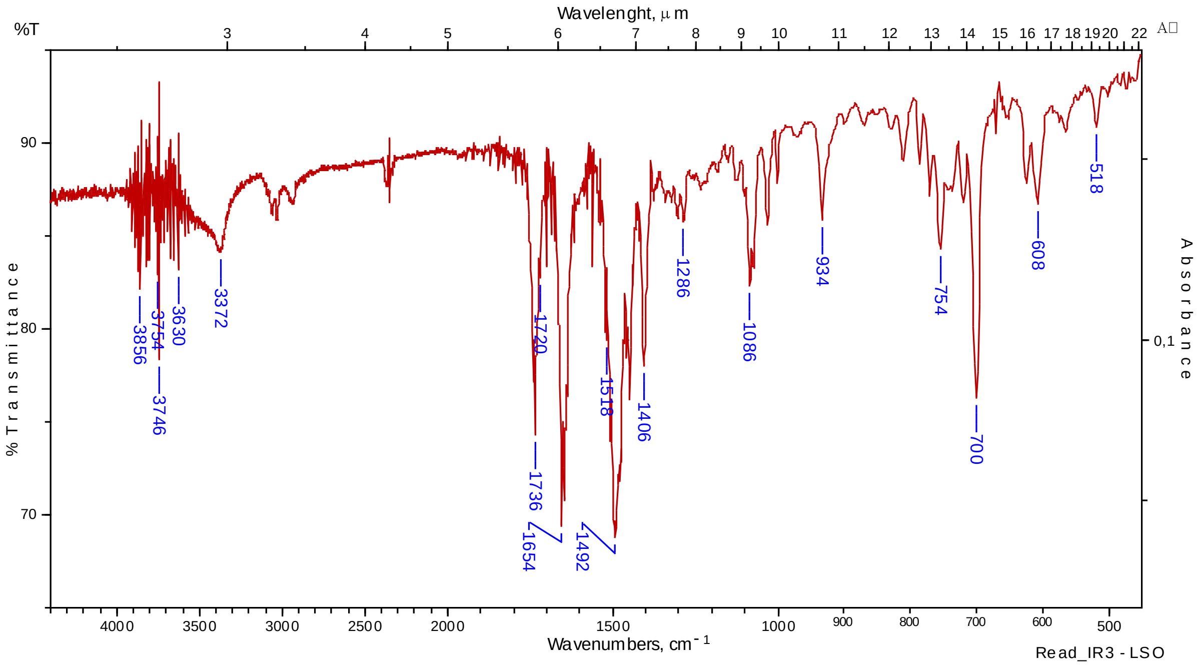

Wavelenght, µ m
%T
% T r a n s m i t t a n c e
A b s o r b a n c e
Wavenumbers, cm⁻¹
Read_IR3 - LSO
3856
3754
3746
3630
3372
1736
1720
1654
1518
1492
1406
1286
1086
934
754
700
608
518

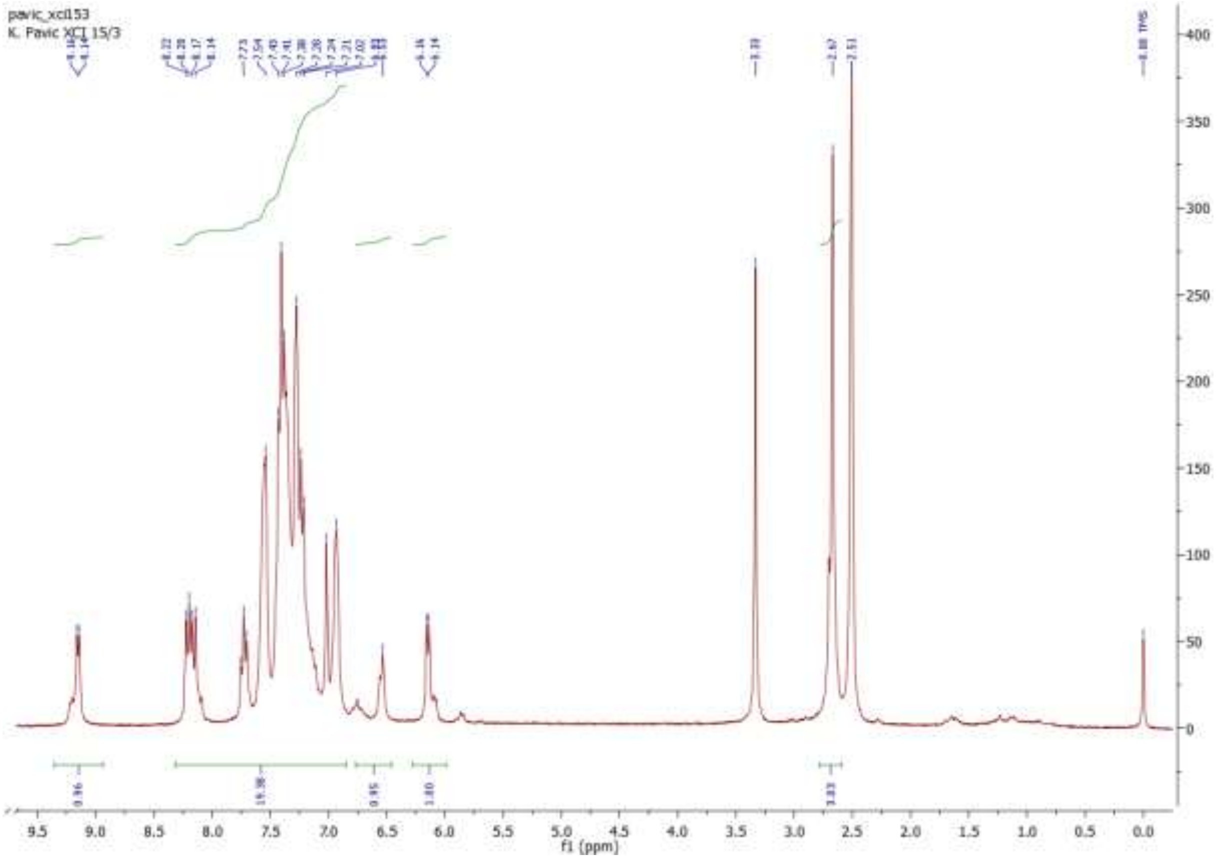

pavic_xcl153
K. Pavic XCI 15/3
f1 (ppm)

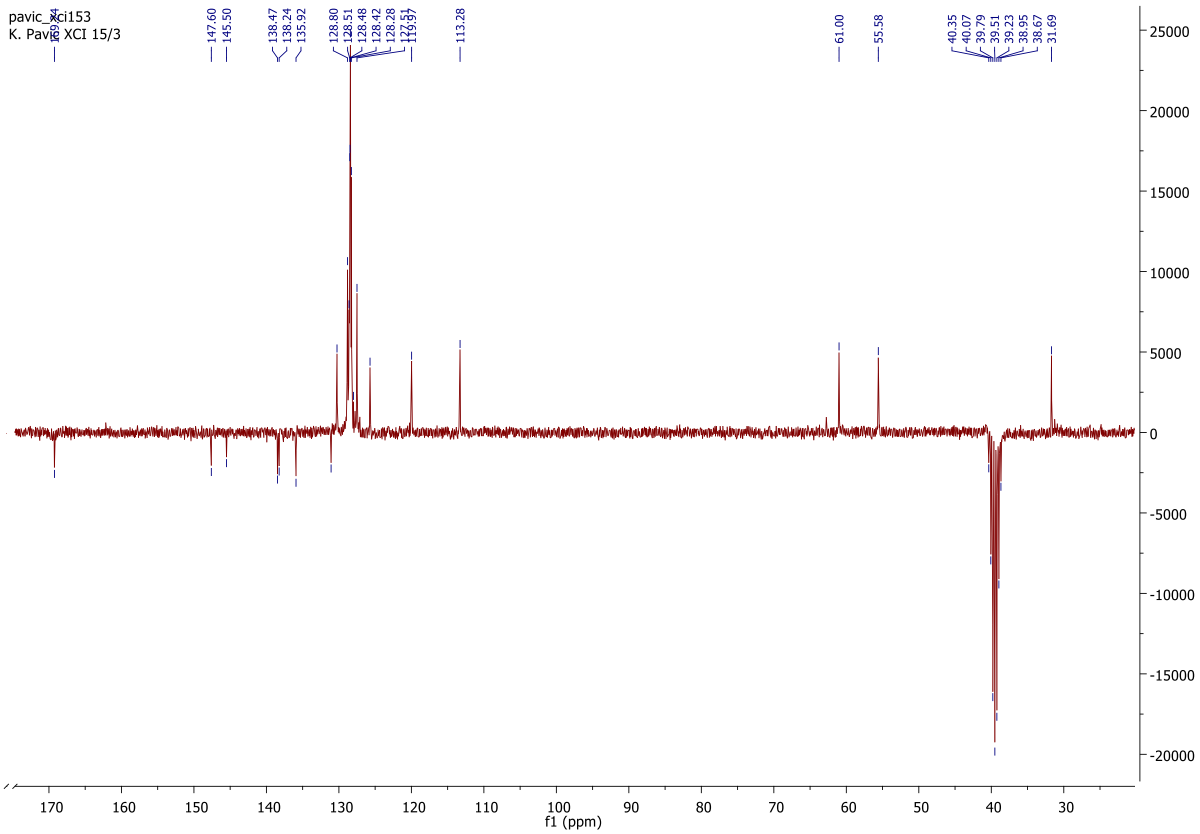
pavic_xci153
K. Pavić XCI 15/3
169.34
147.60
145.50
138.47
138.24
135.92
128.80
128.51
128.48
128.42
128.28
127.51
119.97
113.28
61.00
55.58
40.35
40.07
39.79
39.51
39.23
38.95
38.67
31.69
f1 (ppm)

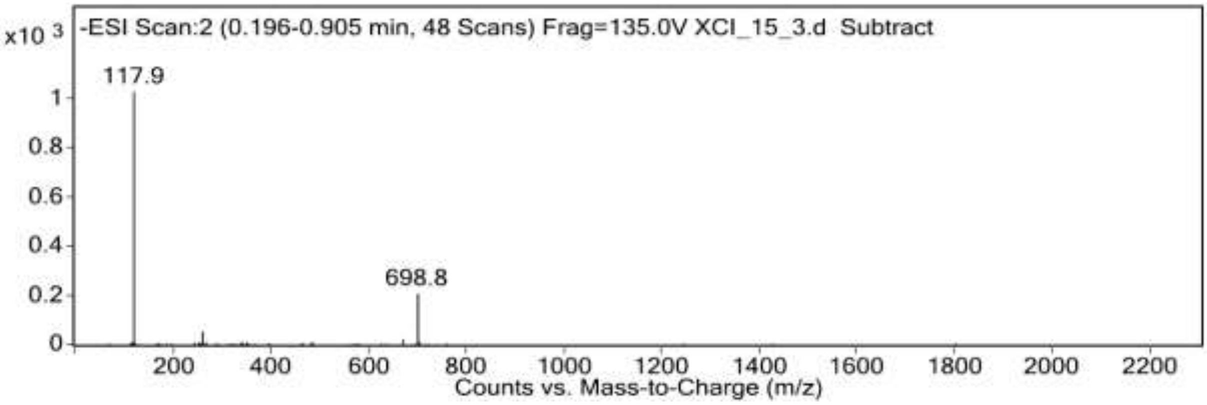
-ESI Scan:2 (0.196-0.905 min, 48 Scans) Frag=135.0V XCI_15_3.d Subtract
x10 3
117.9
698.8
Counts vs. Mass-to-Charge (m/z)

4c

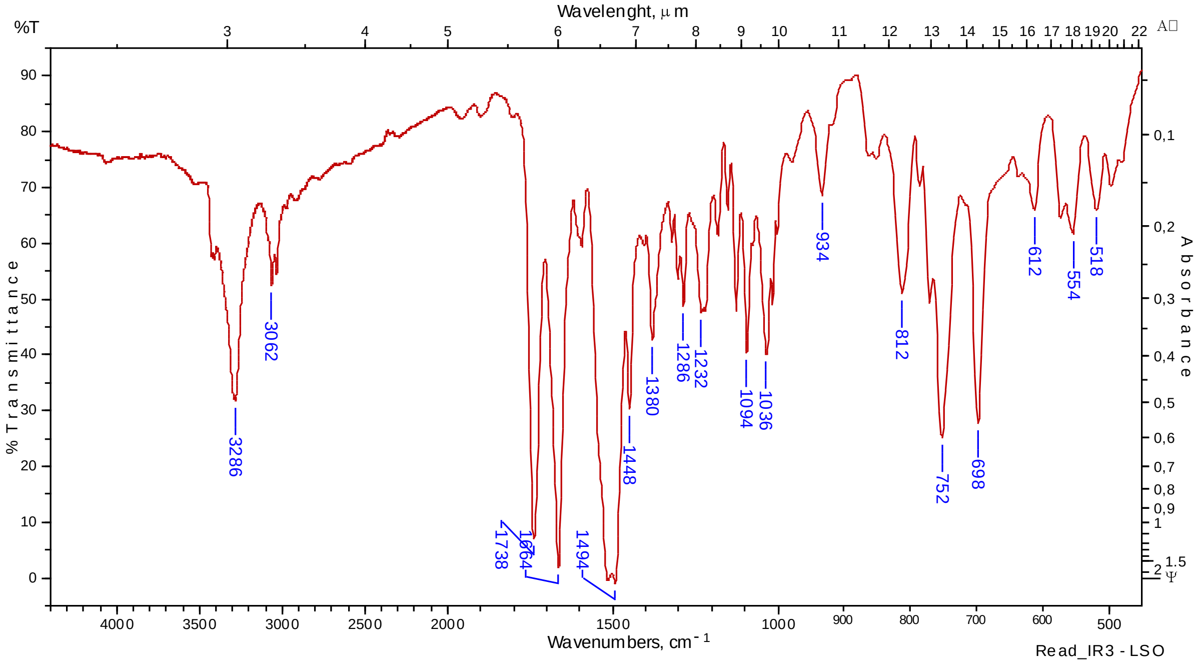
Wavelenght, μ m
%T
% Transmittance
Absorbance
Wavenumbers, cm⁻¹
Read_IR3 - LSO
3286
3062
1738
1664
1494
1448
1380
1286
1232
1094
1036
934
812
752
698
612
554
518

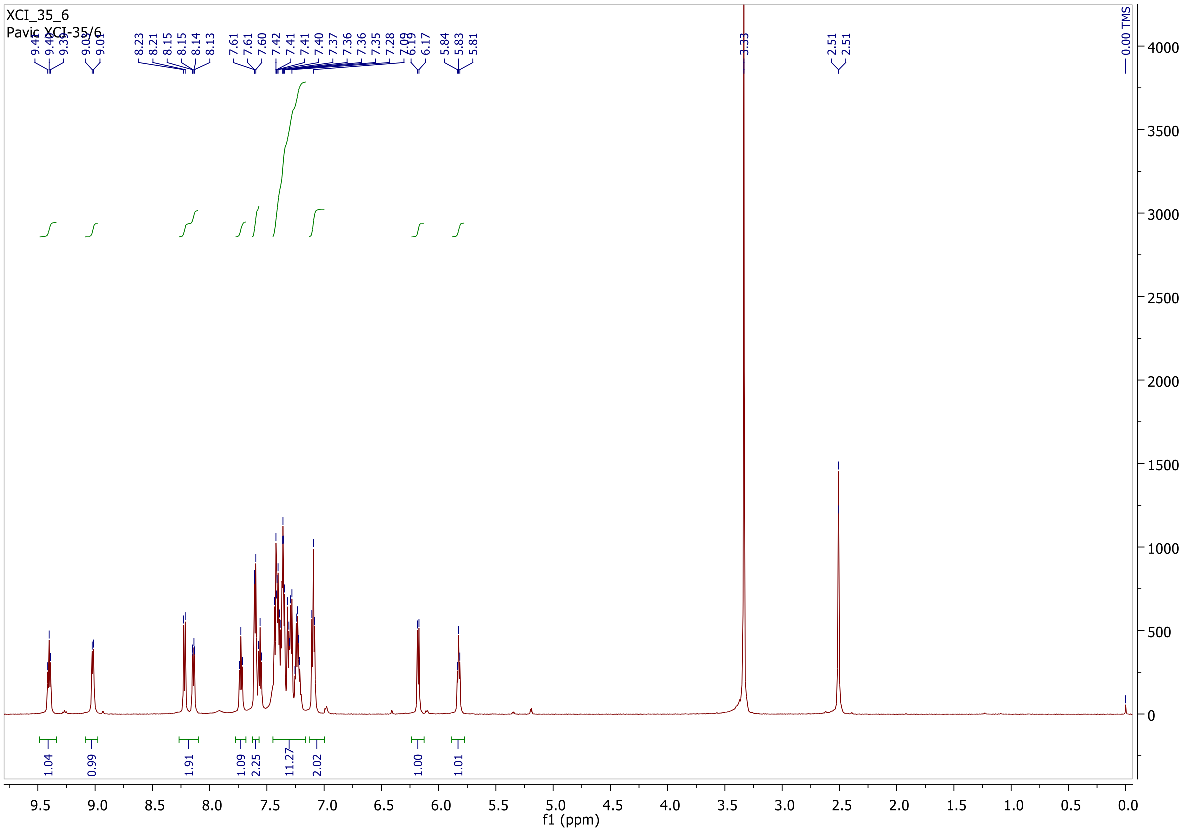
XCI_35_6
Pavic XCI-35/6
3.33
2.51
2.51
0.00 TMS
1.04
0.99
1.91
1.09
2.25
11.27
2.02
1.00
1.01
f1 (ppm)

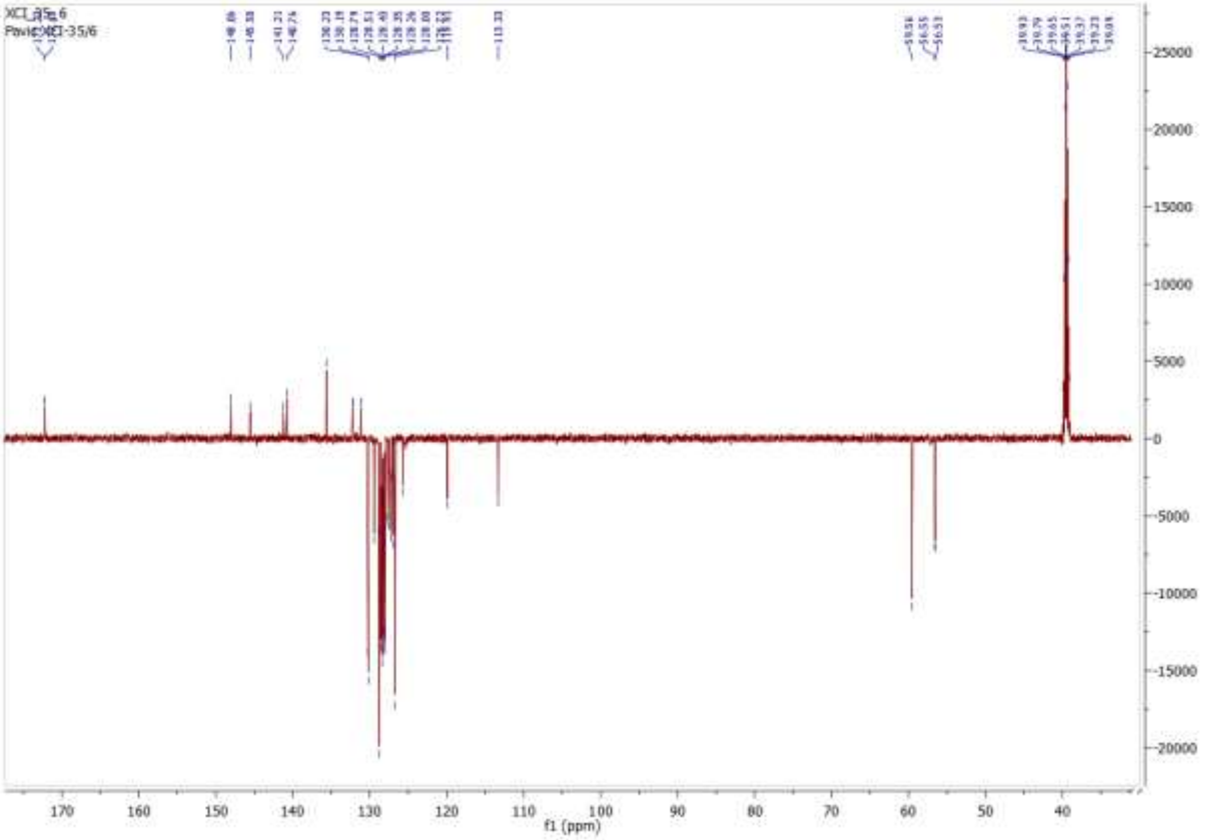
f1 (ppm)

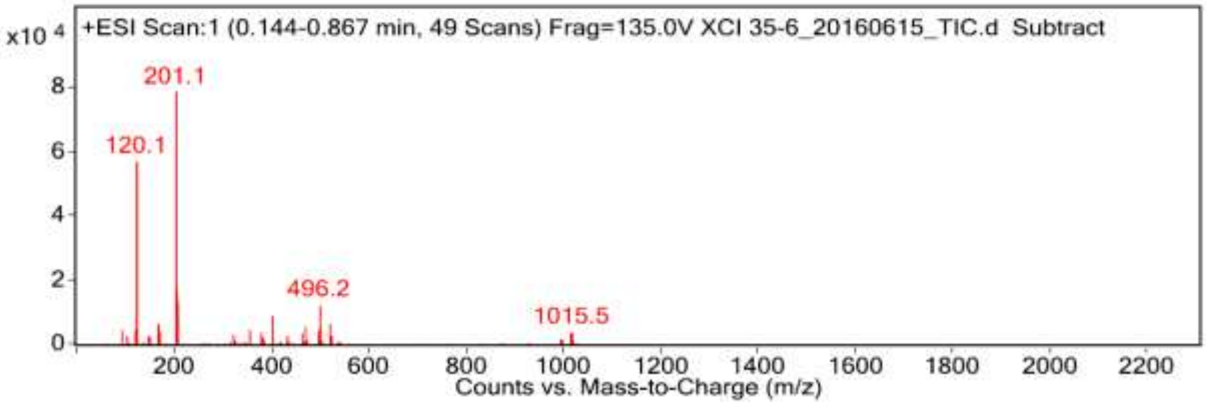
+ESI Scan:1 (0.144-0.867 min, 49 Scans) Frag=135.0V XCI 35-6_20160615_TIC.d Subtract
x10 4
201.1
120.1
496.2
1015.5
Counts vs. Mass-to-Charge (m/z)

4d

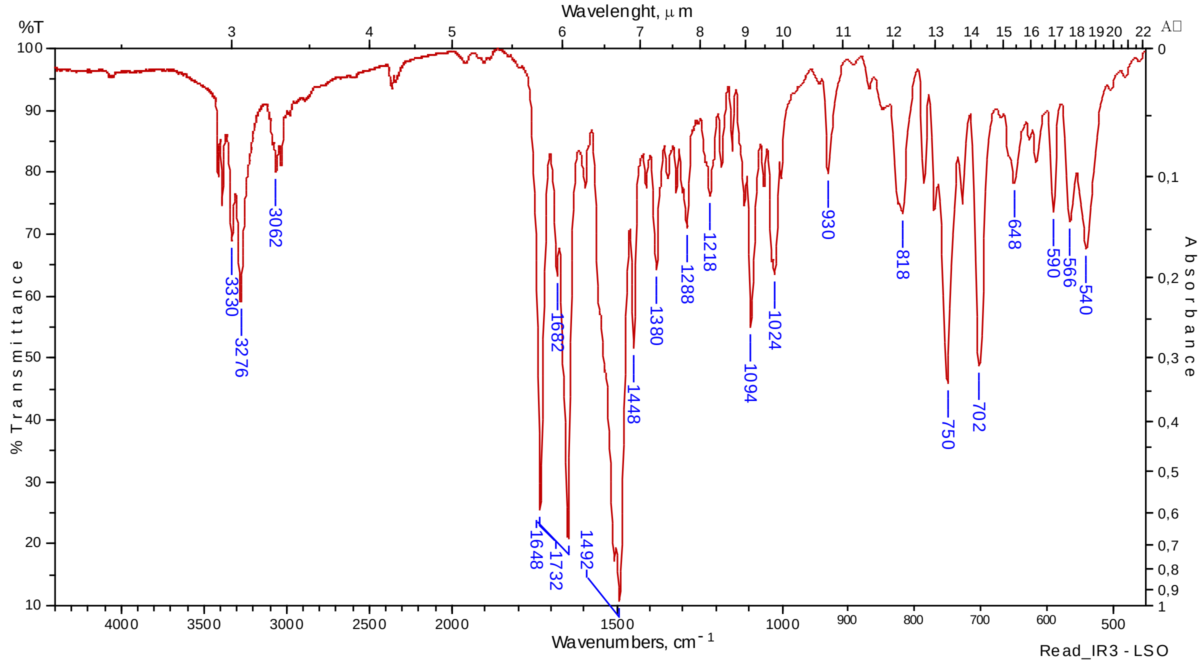

Wavelenght, µ m
%T
Wavenumbers, cm⁻¹
% T r a n s m i t t a n c e
A b s o r b a n c e
3330
3276
3062
1648
1732
1682
1492
1448
1380
1288
1218
1094
1024
930
818
750
702
648
590
566
540
Read_IR3 - LSO

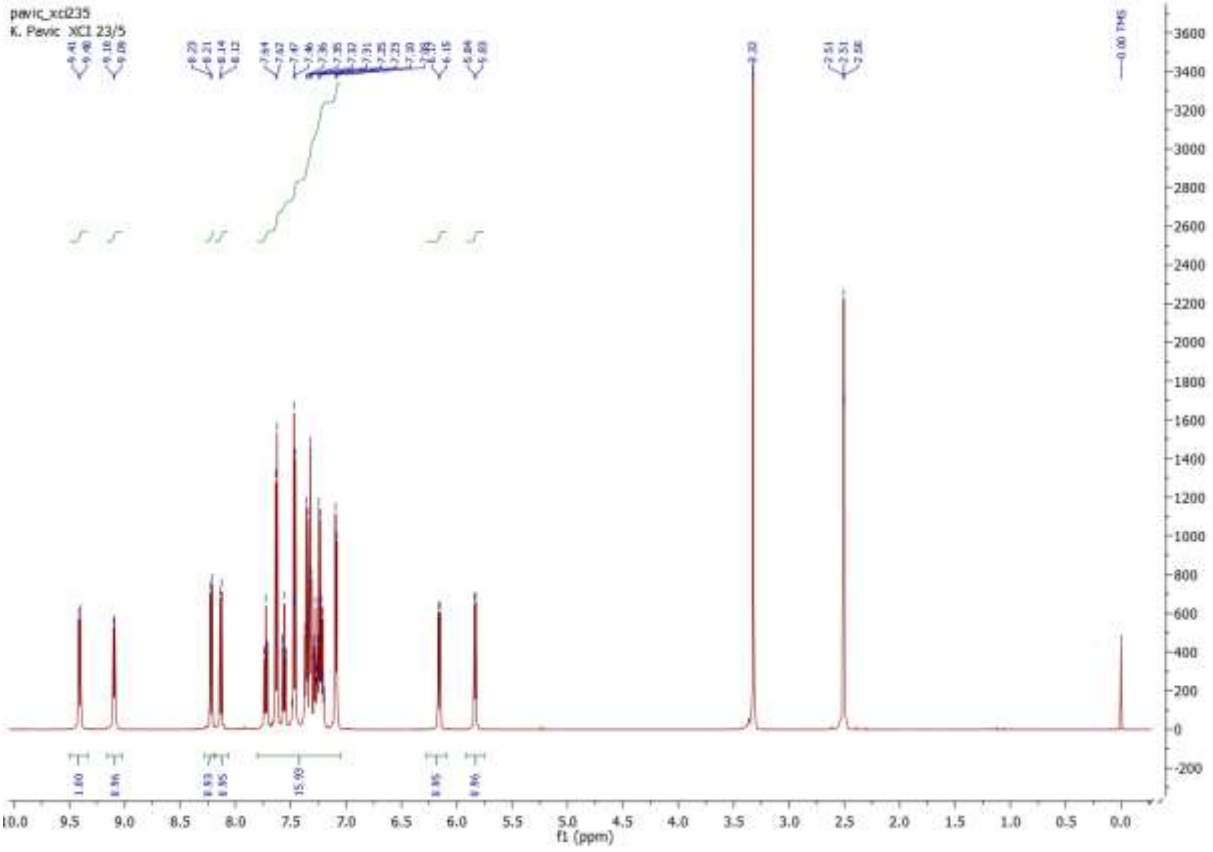

pavic_xci235
K. Pavic XCI 23/5
f1 (ppm)

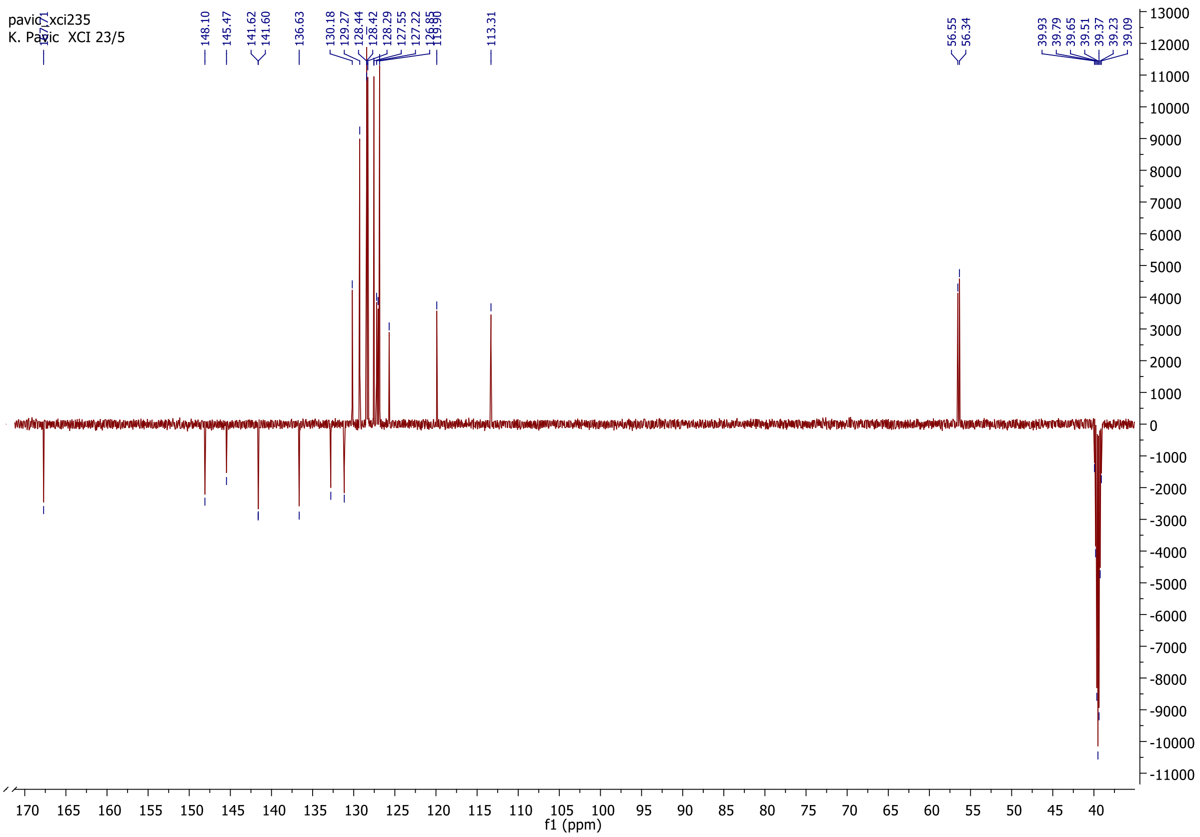
pavic_xci235
K. Pavic XCI 23/5
148.10
145.47
141.62
141.60
136.63
130.18
129.27
128.44
128.42
128.29
127.55
127.22
113.31
56.55
56.34
39.93
39.79
39.65
39.51
39.37
39.23
39.09
f1 (ppm)

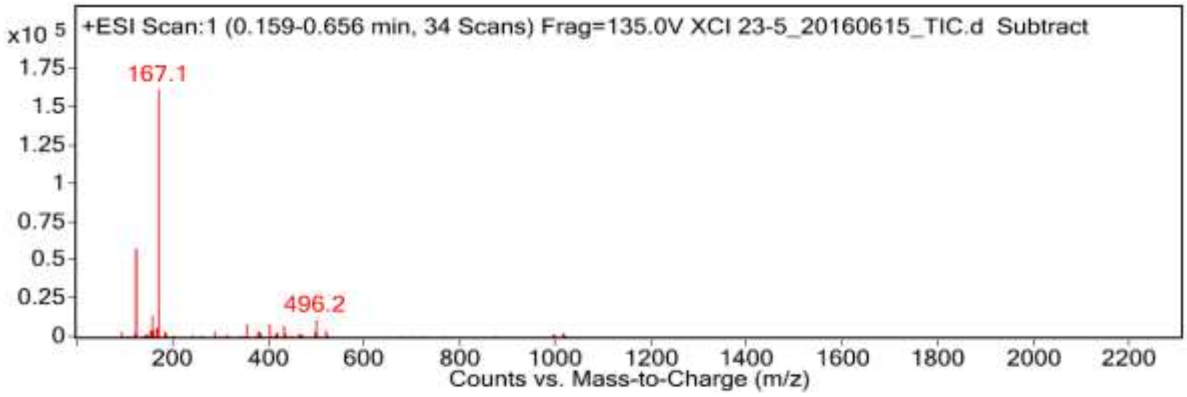
+ESI Scan:1 (0.159-0.656 min, 34 Scans) Frag=135.0V XCI 23-5_20160615_TIC.d Subtract
x10 5
167.1
496.2
Counts vs. Mass-to-Charge (m/z)

4e

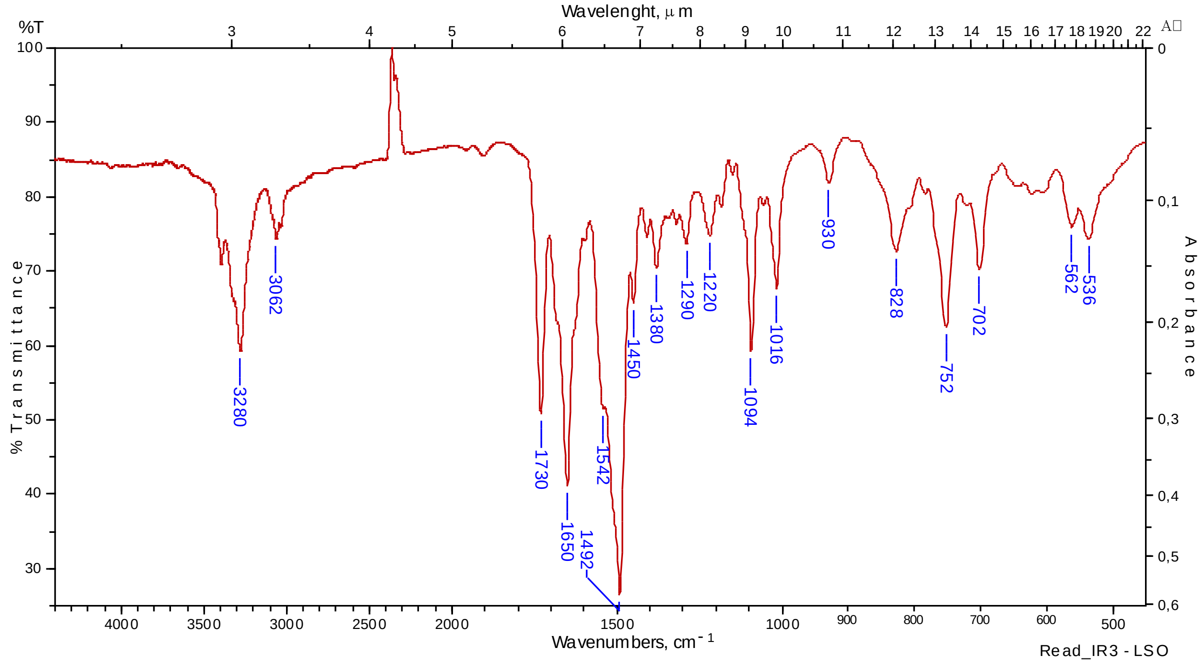

Wavelenght, μ m
%T
A
% Transmittance
Absorbance
Wavenumbers, cm⁻¹
Read_IR3 - LSO
3280
3062
1730
1650
1542
1492
1450
1380
1290
1220
1094
1016
930
828
752
702
562
536

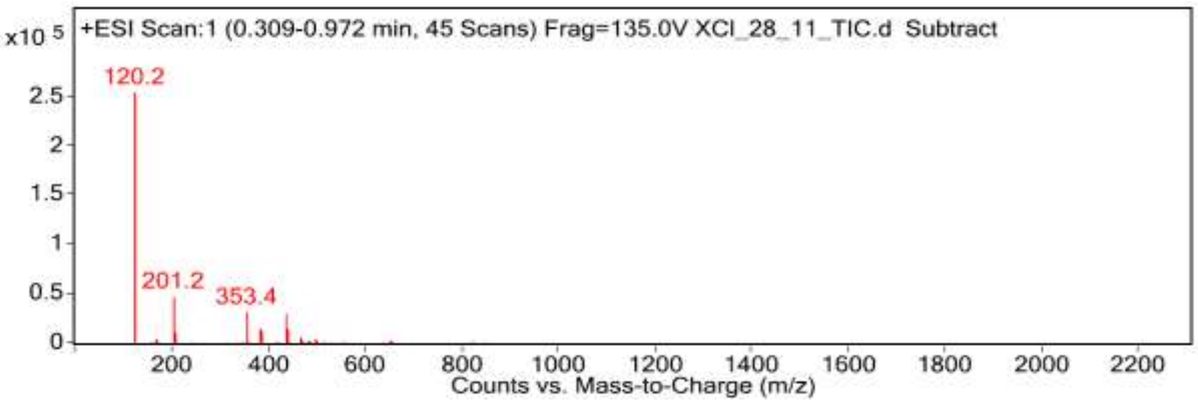

x10 5
+ESI Scan:1 (0.309-0.972 min, 45 Scans) Frag=135.0V XCI_28_11_TIC.d Subtract
120.2
201.2
353.4
Counts vs. Mass-to-Charge (m/z)

4f

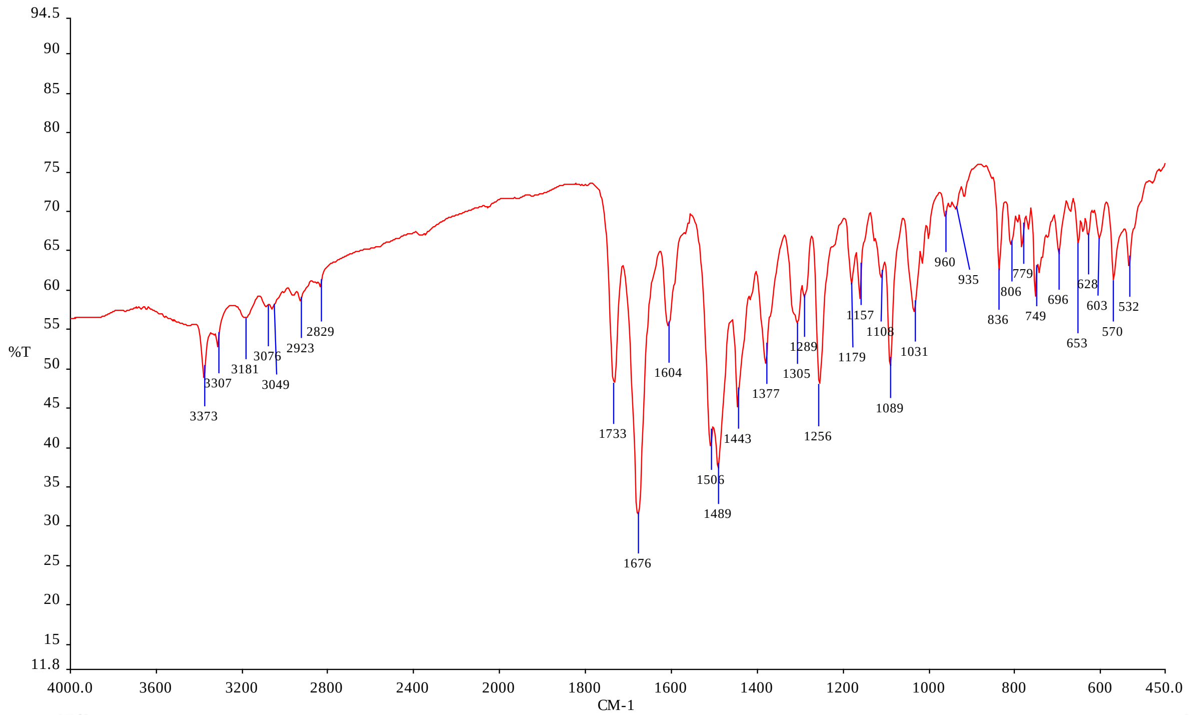
%T
CM-1
3373
3307
3181
3076
3049
2923
2829
1733
1676
1604
1506
1489
1443
1377
1305
1289
1256
1179
1157
1108
1089
1031
960
935
836
806
779
749
696
653
628
603
570
532

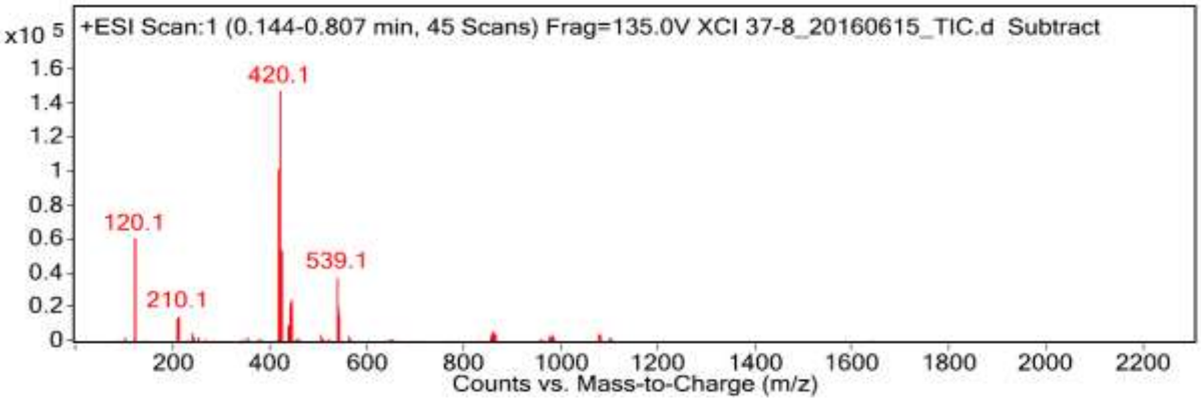
+ESI Scan:1 (0.144-0.807 min, 45 Scans) Frag=135.0V XCI 37-8_20160615_TIC.d Subtract
x10 5
420.1
120.1
539.1
210.1
Counts vs. Mass-to-Charge (m/z)

5a

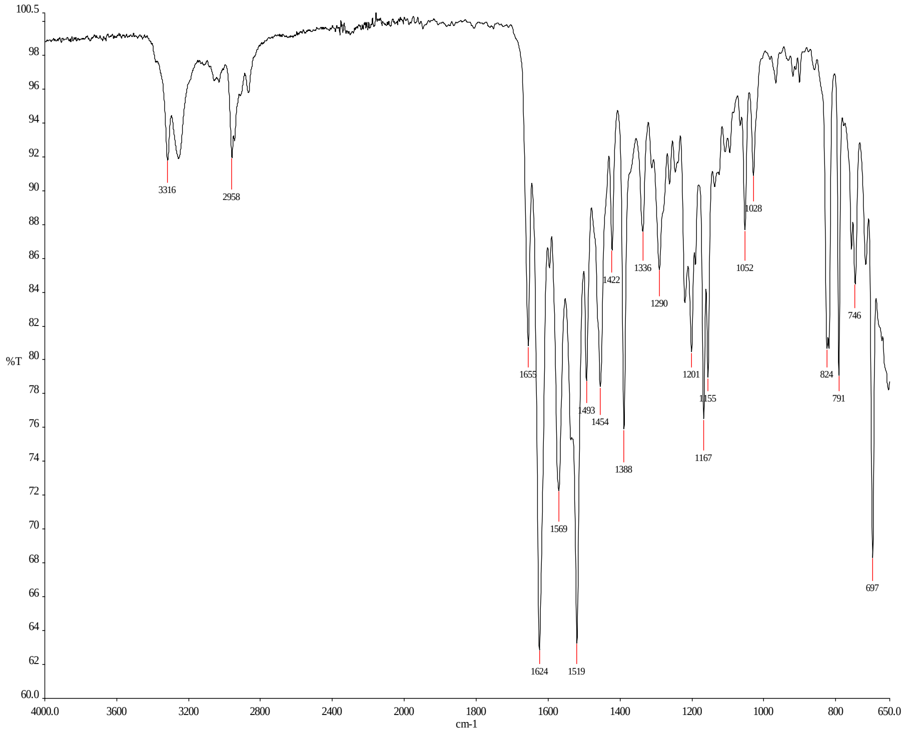

%T
cm-1
3316
2958
1655
1624
1569
1519
1493
1454
1422
1388
1336
1290
1201
1167
1155
1052
1028
824
791
746
697

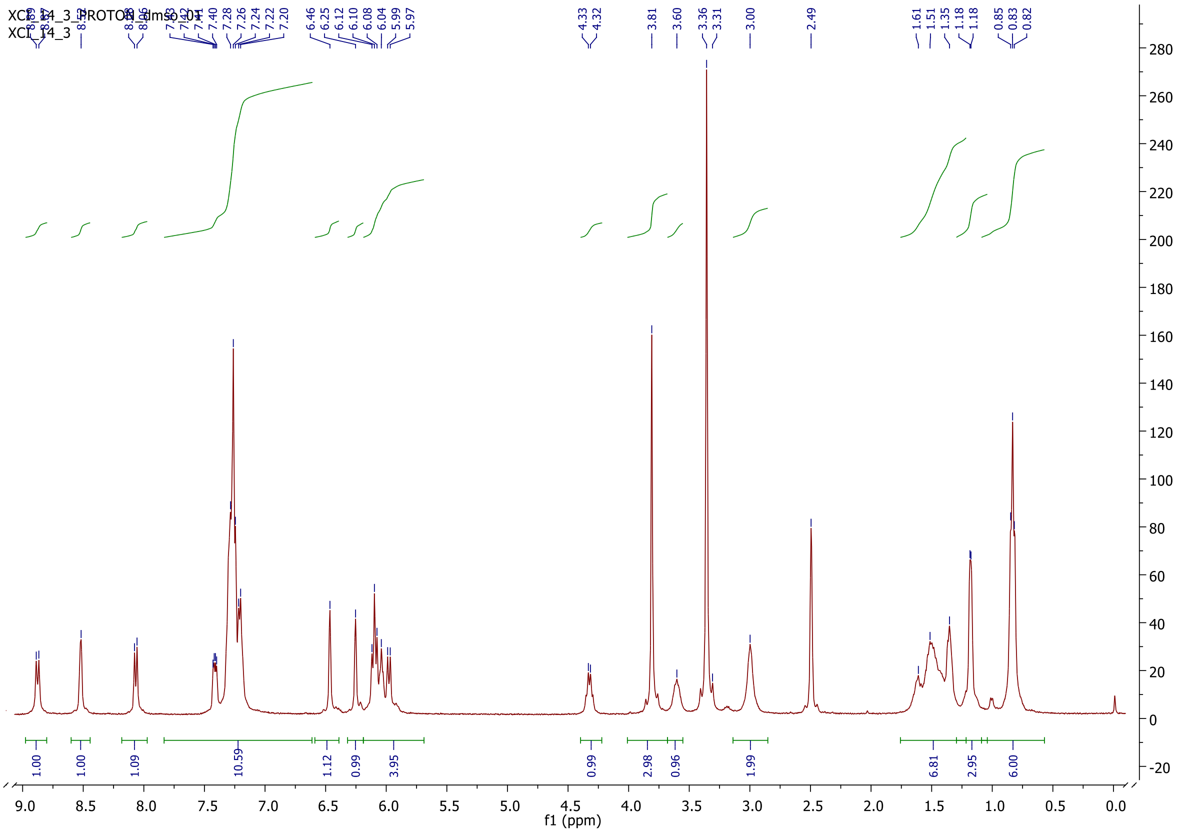

XCL_14_3_PROTON_dmso_01
XCL_14_3
f1 (ppm)

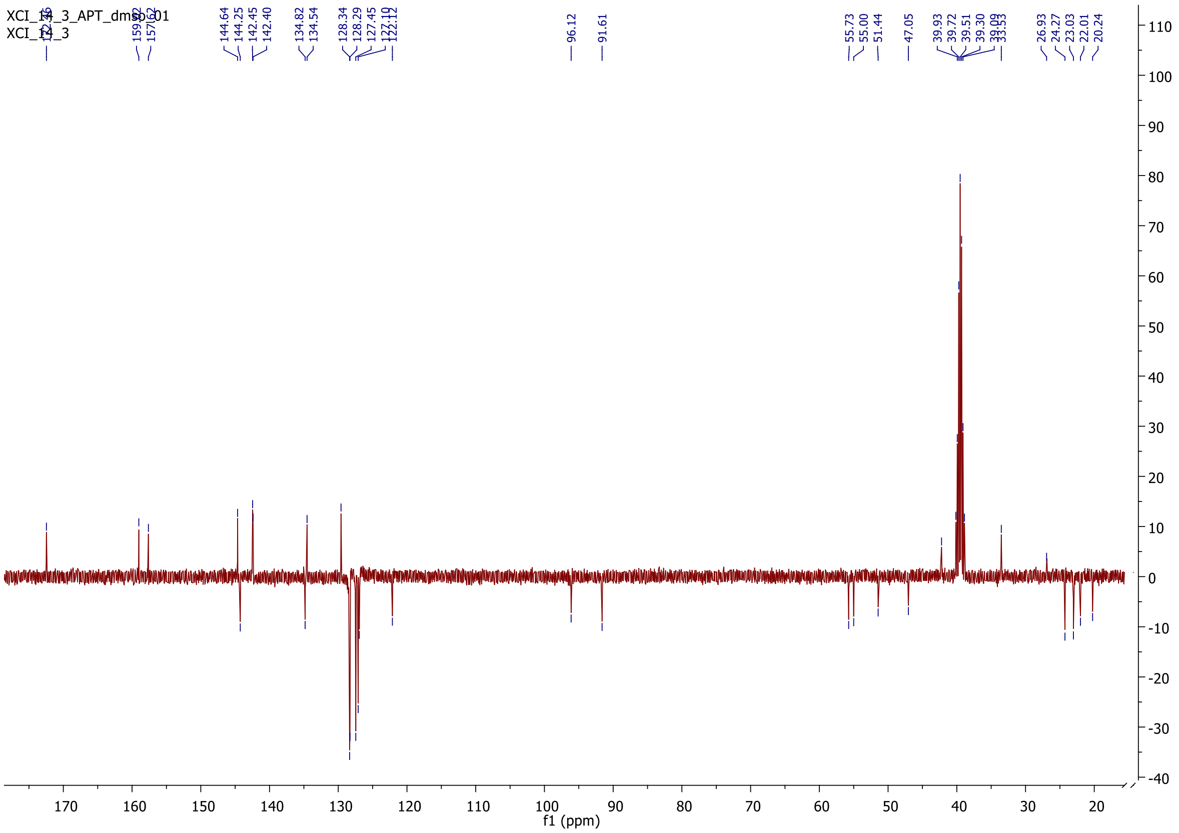
XCI_14_3_APT_dmso_01
XCI_14_3
159.92
157.62
144.64
144.25
142.45
142.40
134.82
134.54
128.34
128.29
127.45
122.12
96.12
91.61
55.73
55.00
51.44
47.05
39.93
39.72
39.51
39.30
33.53
26.93
24.27
23.03
22.01
20.24
f1 (ppm)

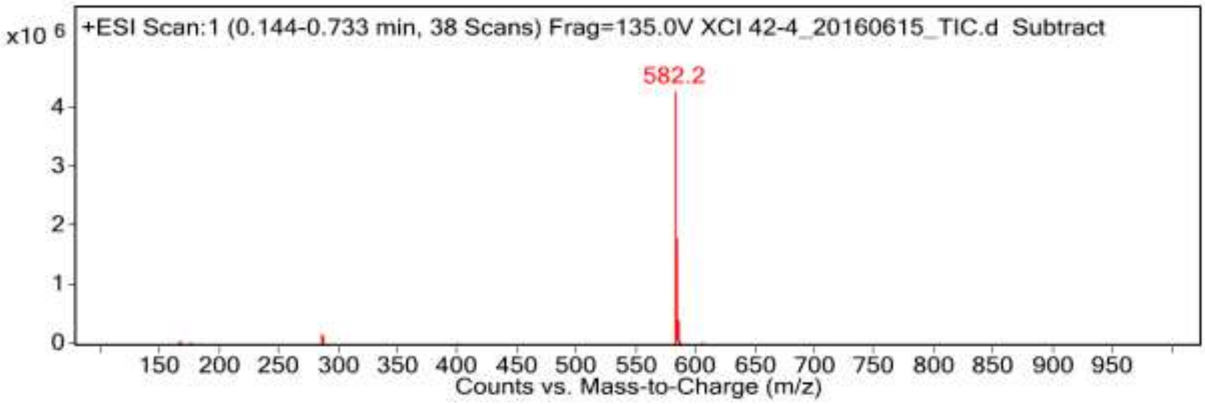
x10 6
+ESI Scan:1 (0.144-0.733 min, 38 Scans) Frag=135.0V XCI 42-4_20160615_TIC.d Subtract
582.2
Counts vs. Mass-to-Charge (m/z)

5b

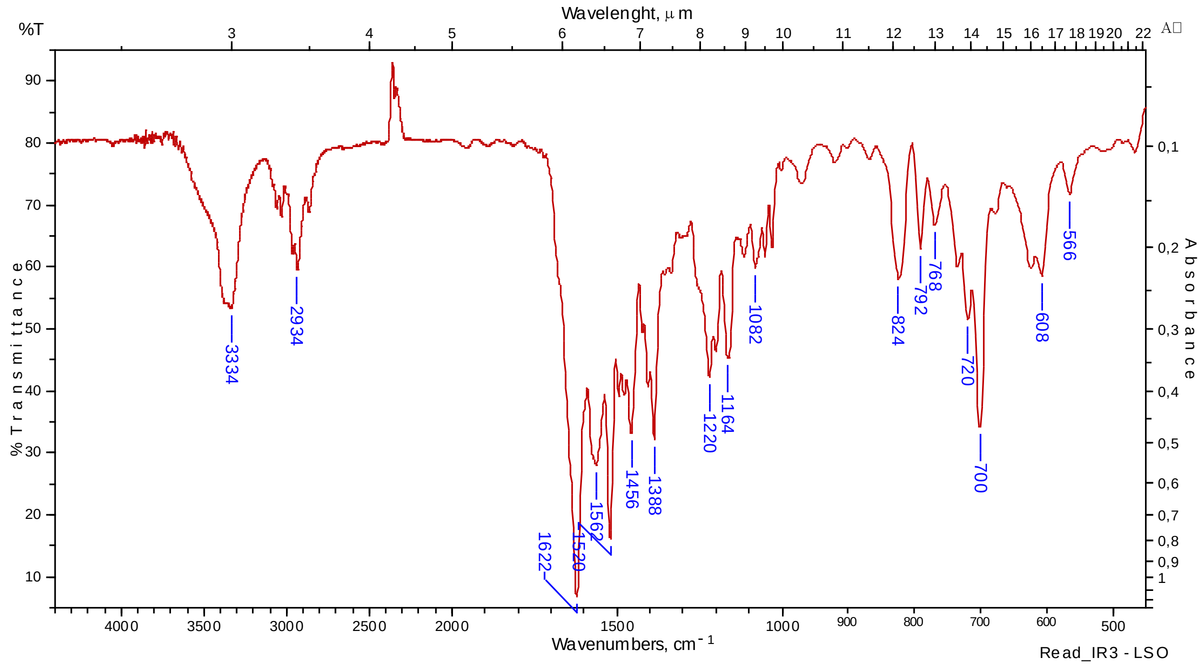

Wavelenght, µ m
%T
% Transmittance
Absorbance
3334
2934
1622
1520
1562
1456
1388
1220
1164
1082
824
792
768
720
700
608
566
Wavenumbers, cm-1
Read_IR3 - LSO

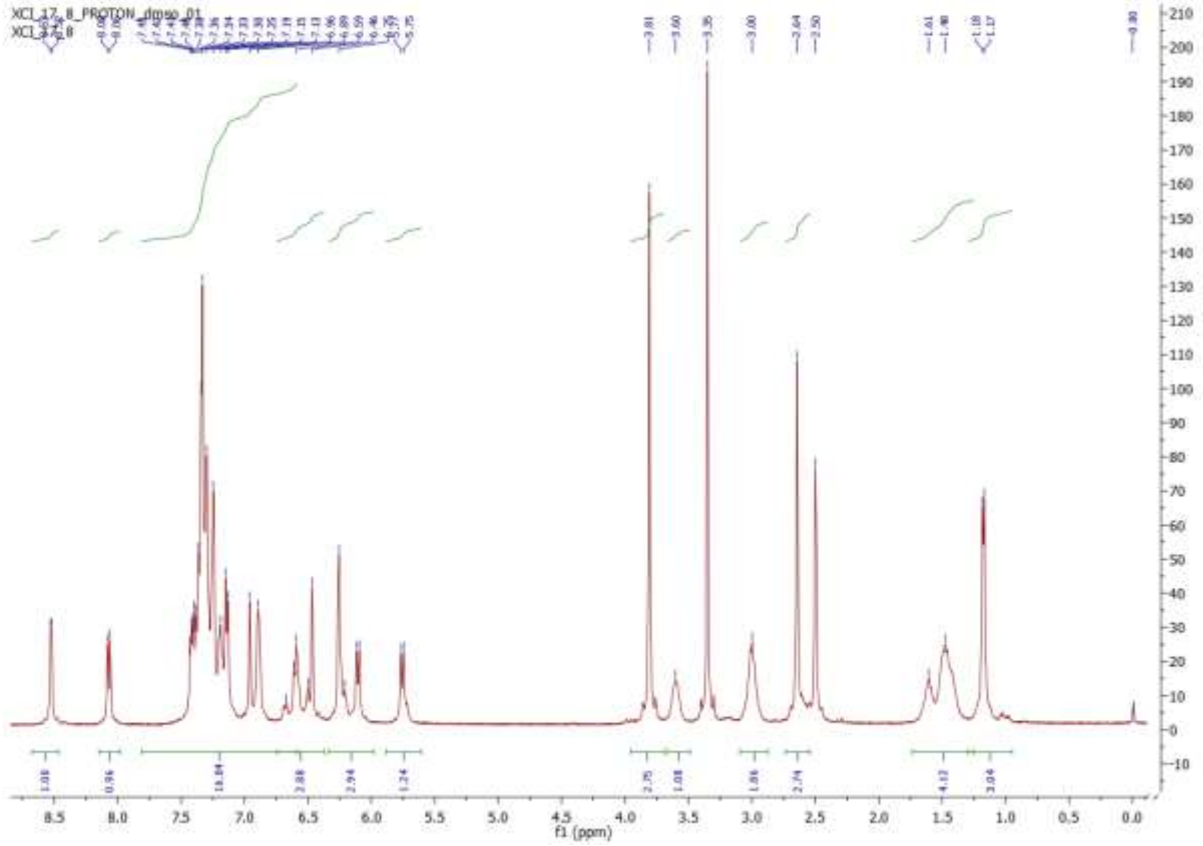

XCI_17_8_PROTON_dmso_01
f1 (ppm)

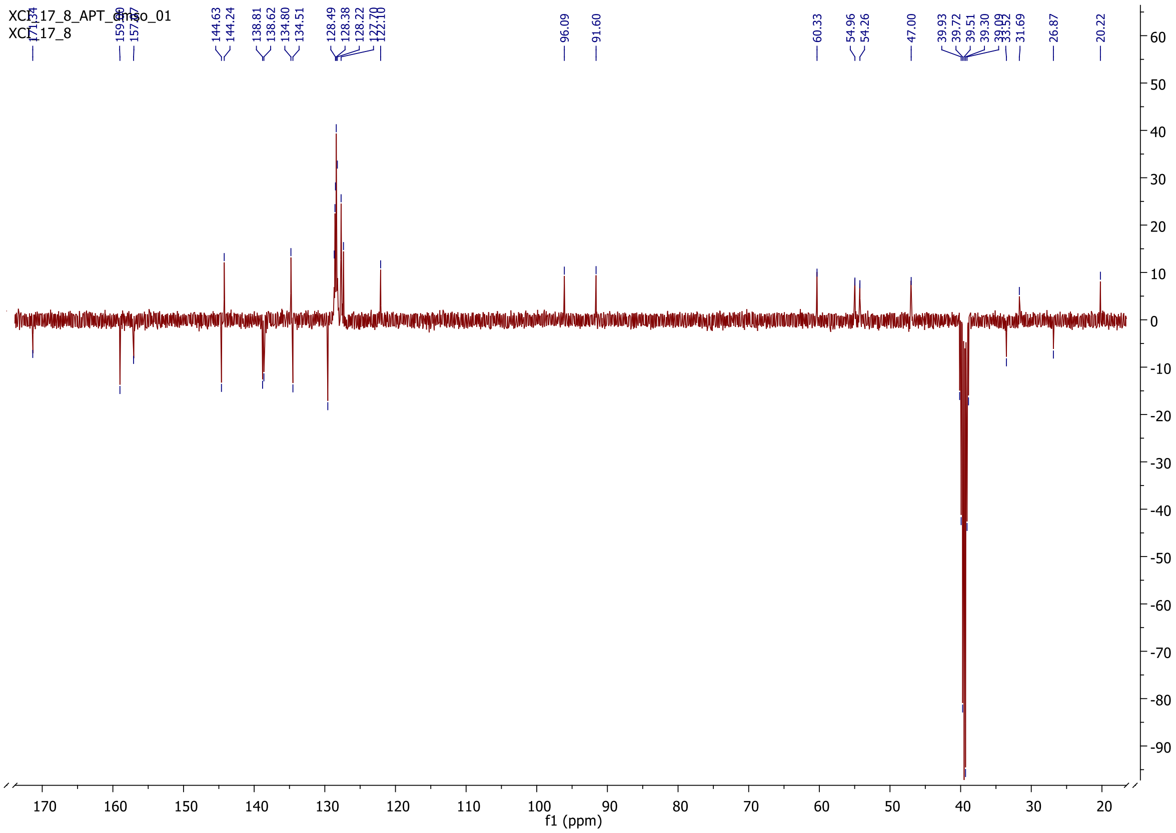

XCI_17_8_APT_dmso_01
XCI_17_8
f1 (ppm)

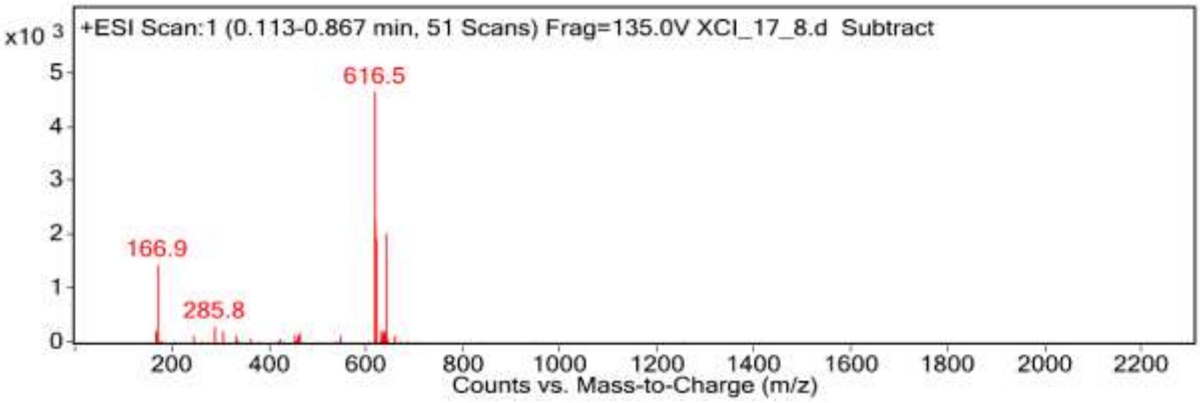

+ESI Scan:1 (0.113-0.867 min, 51 Scans) Frag=135.0V XCI_17_8.d Subtract
x10 3
616.5
166.9
285.8
Counts vs. Mass-to-Charge (m/z)

5c

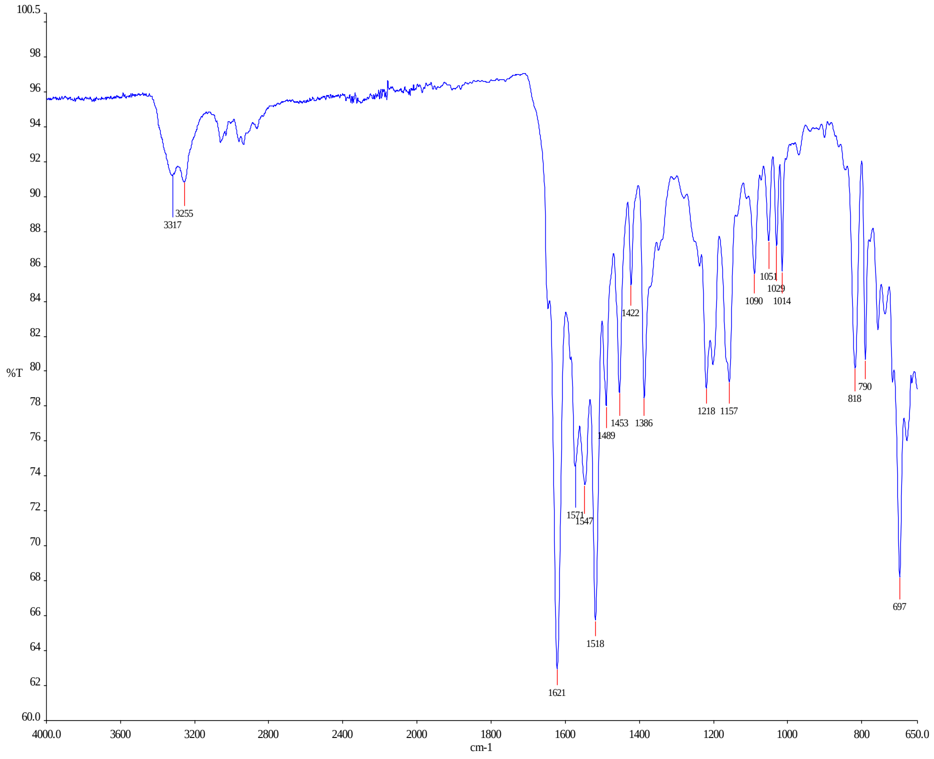
100.5
98
96
94
92
90
88
86
84
82
80
78
76
74
72
70
68
66
64
62
60.0
%T
4000.0
3600
3200
2800
2400
2000
1800
1600
1400
1200
1000
800
650.0
cm-1
3255
3317
1422
1453
1386
1489
1571
1547
1518
1621
1051
1029
1090
1014
1218
1157
790
818
697

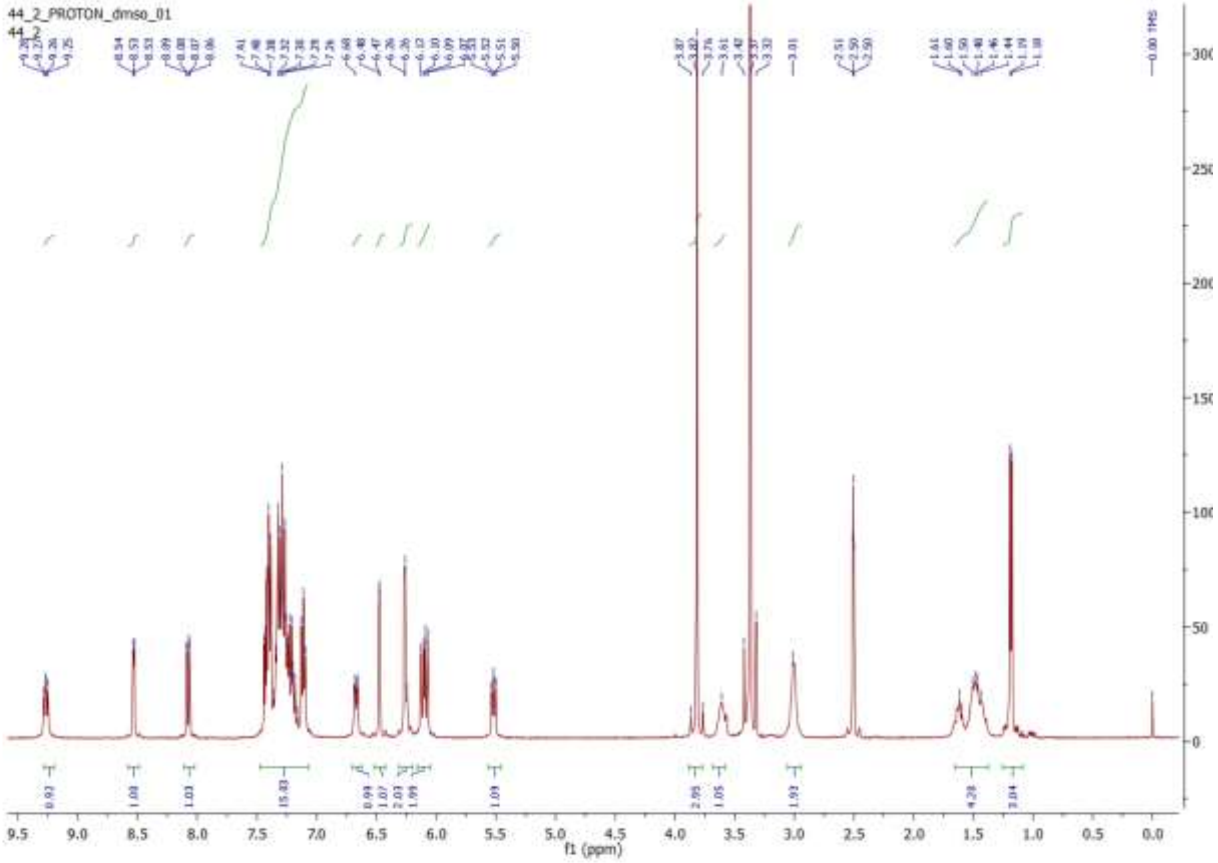
44_2_PROTON_dmso_01
44_2
f1 (ppm)

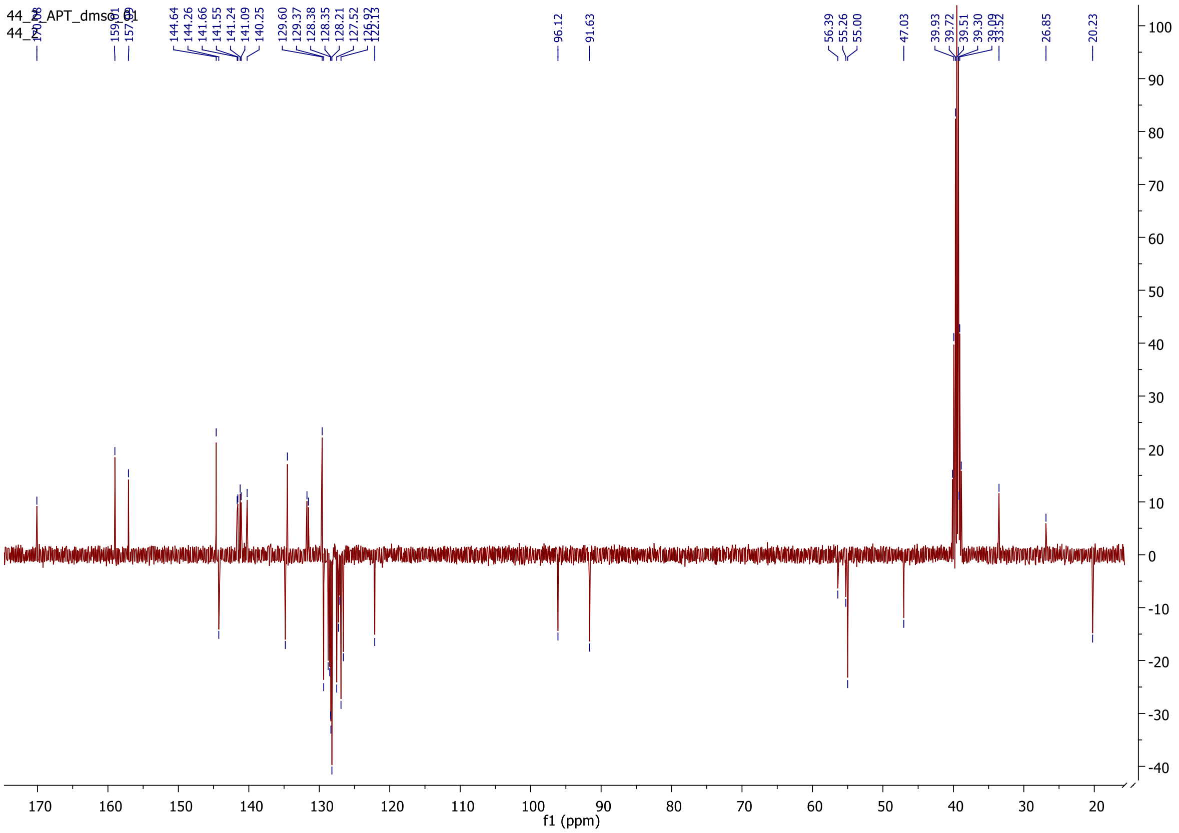
44_2_APT_dmso.01
f1 (ppm)

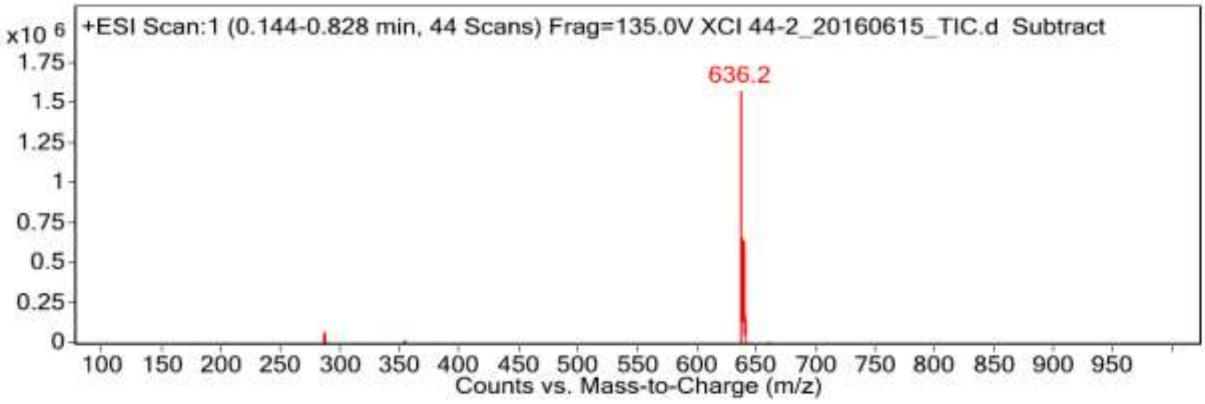
+ESI Scan:1 (0.144-0.828 min, 44 Scans) Frag=135.0V XCI 44-2_20160615_TIC.d Subtract
636.2
Counts vs. Mass-to-Charge (m/z)

5d

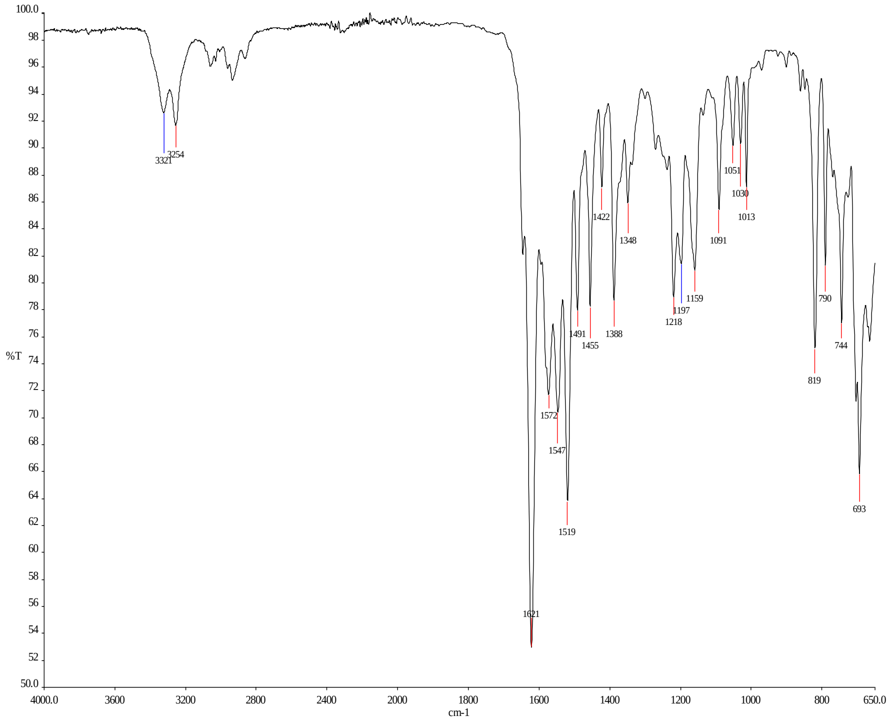
%T
cm-1
3321
3254
1621
1572
1547
1519
1491
1455
1422
1388
1348
1218
1197
1159
1091
1051
1030
1013
819
790
744
693

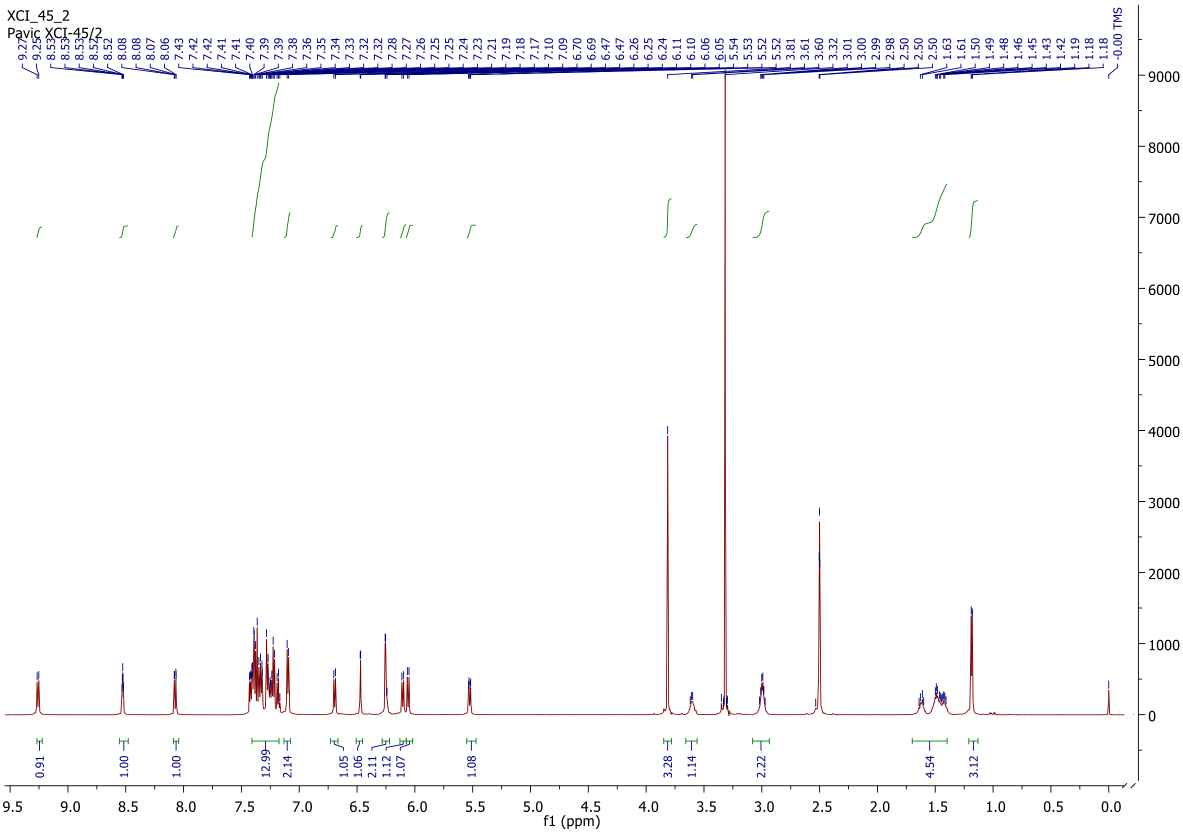
XCI_45_2
Pavic XCI-45/2
9.27 9.25 8.53 8.53 8.53 8.52 8.52 8.08 8.08 8.07 8.06 7.43 7.42 7.42 7.41 7.41 7.40 7.39 7.39 7.38 7.36 7.35 7.34 7.33 7.32 7.32 7.28 7.27 7.26 7.25 7.25 7.24 7.23 7.21 7.19 7.18 7.17 7.10 7.09 6.70 6.69 6.47 6.47 6.26 6.25 6.24 6.11 6.10 6.06 6.05 5.54 5.53 5.52 5.52 3.81 3.61 3.60 3.32 3.01 3.00 2.99 2.98 2.50 2.50 1.63 1.61 1.50 1.49 1.48 1.46 1.45 1.43 1.42 1.19 1.18 -0.00 TMS
0.91 1.00 1.00 12.99 2.14 1.05 1.06 2.11 1.12 1.07 1.08 3.28 1.14 2.22 4.54 3.12
f1 (ppm)

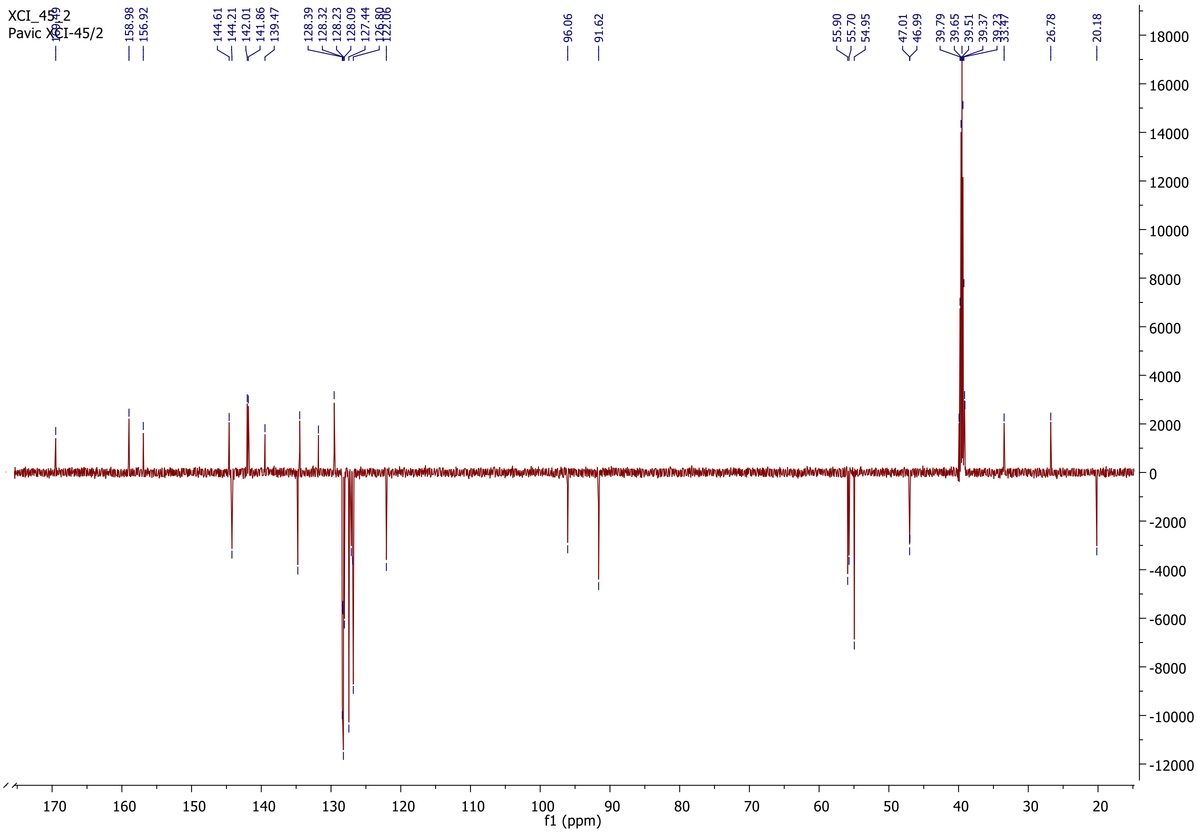

XCI_45_2
Pavic XCI-45/2
f1 (ppm)

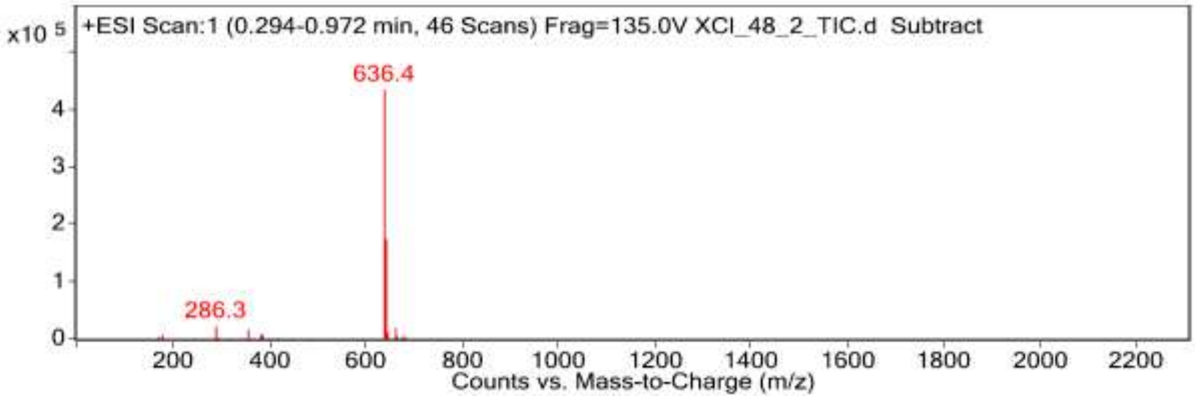

+ESI Scan:1 (0.294-0.972 min, 46 Scans) Frag=135.0V XCI_48_2_TIC.d Subtract
x10 5
636.4
286.3
Counts vs. Mass-to-Charge (m/z)

5e

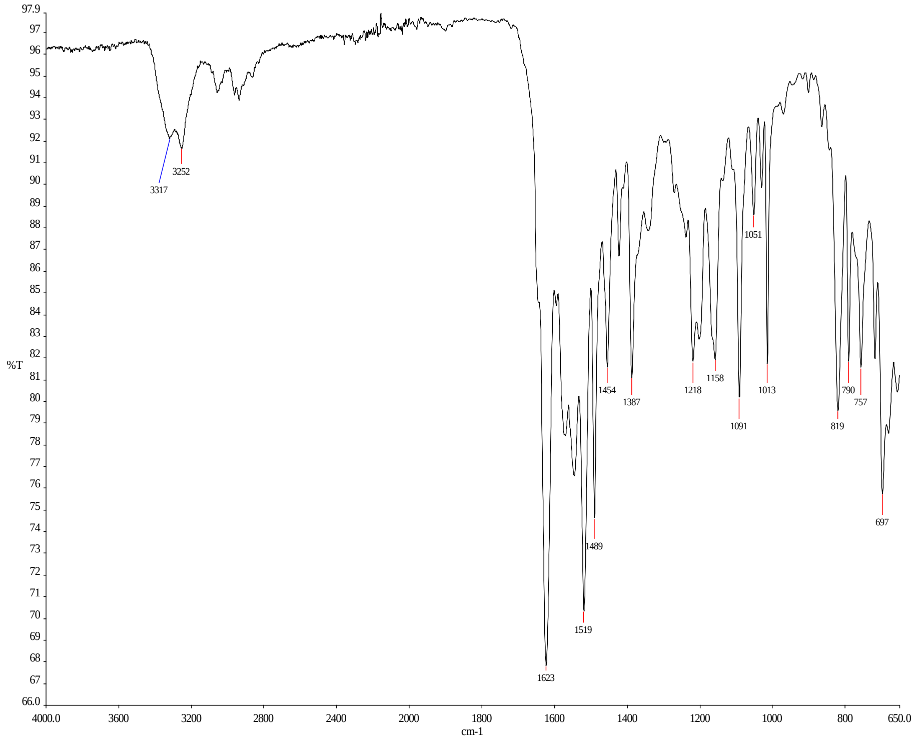
%T
cm-1
3317
3252
1623
1519
1489
1454
1387
1218
1158
1091
1051
1013
819
790
757
697

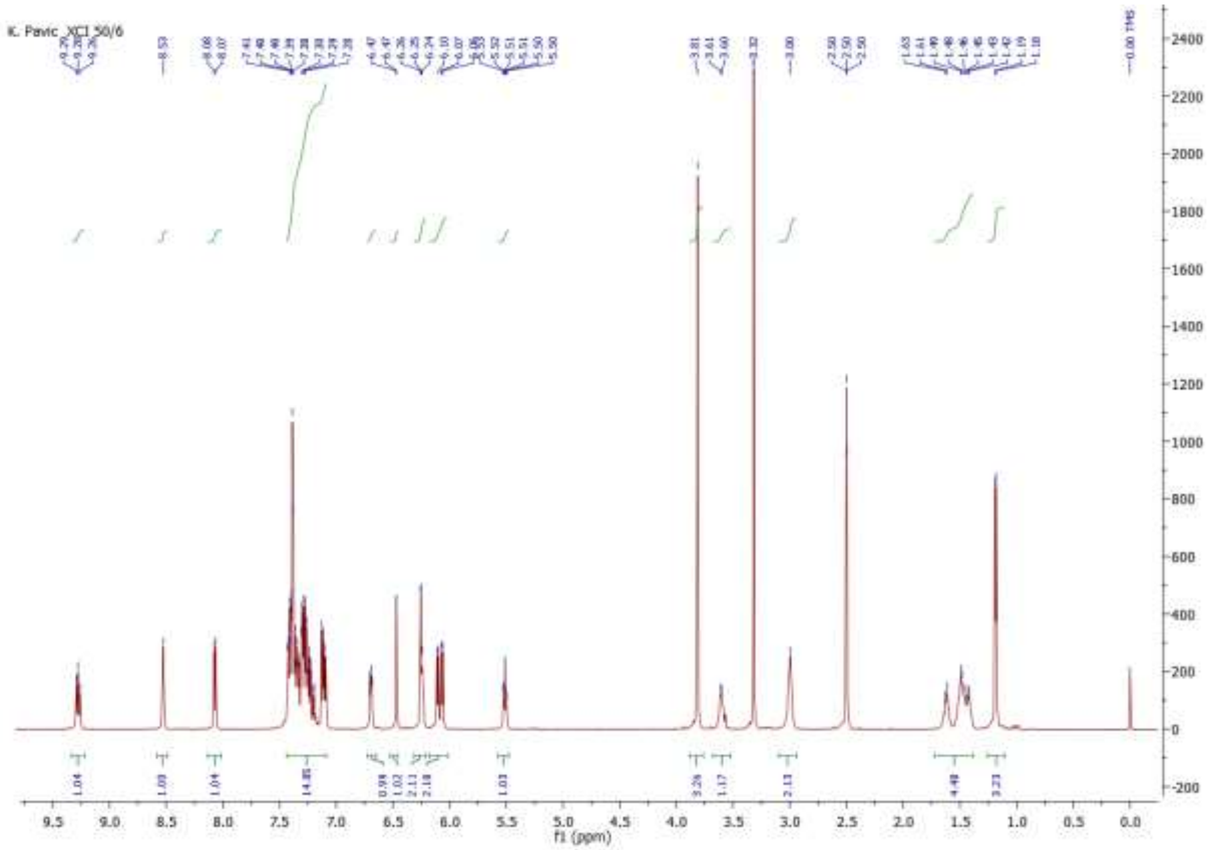
K. Pavic_XCI 50/6
f1 (ppm)

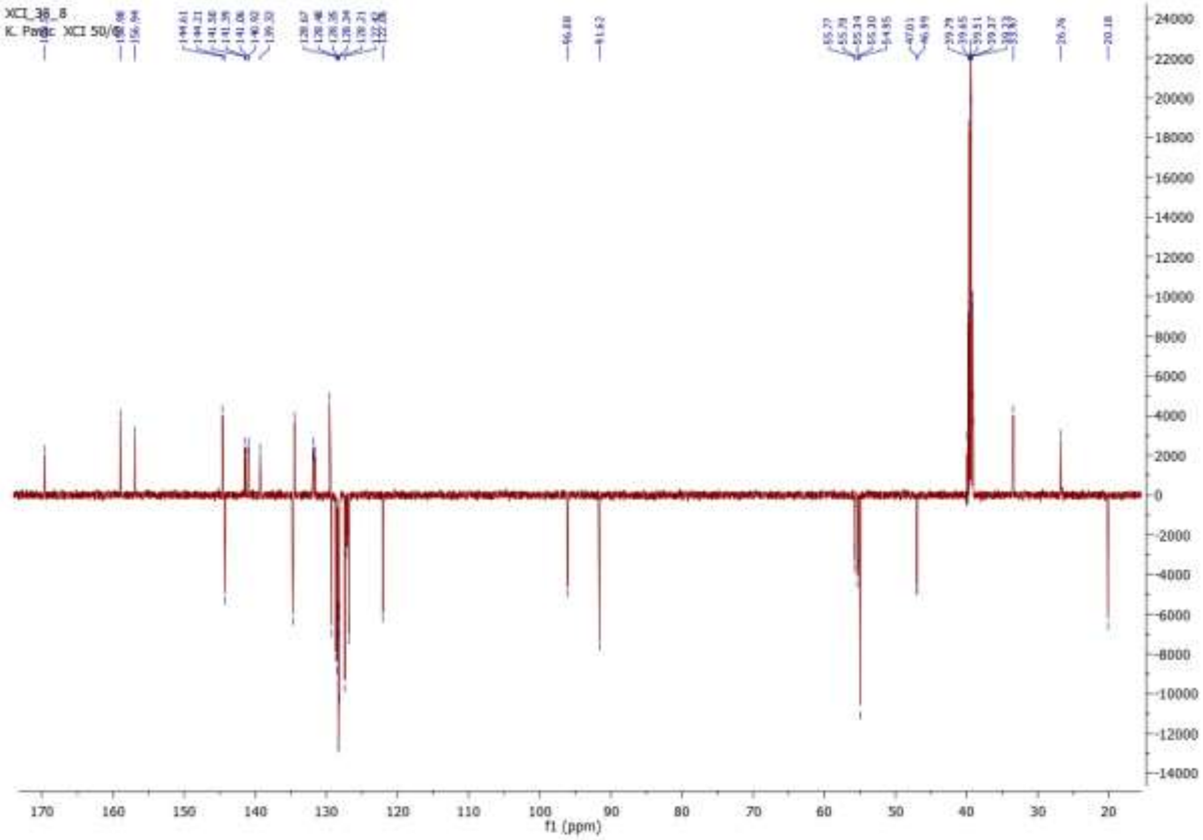
f1 (ppm)

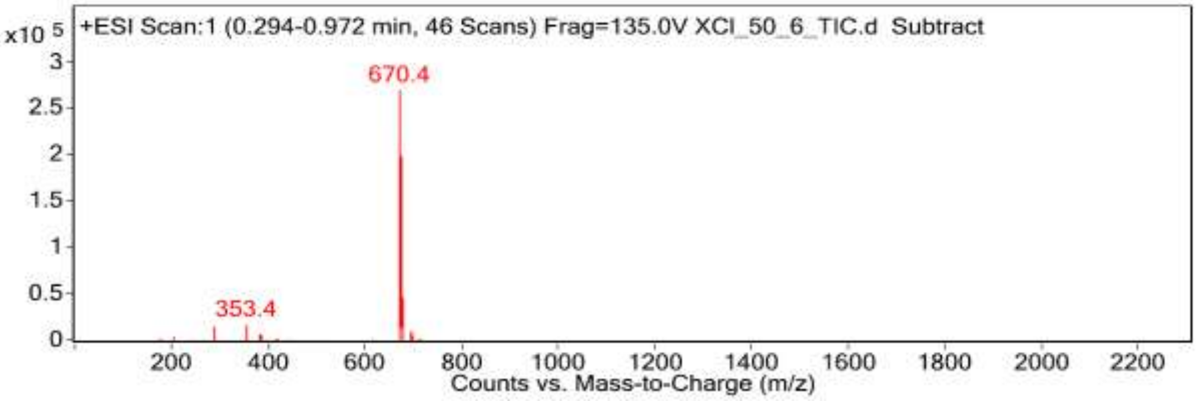
x10 5
+ESI Scan:1 (0.294-0.972 min, 46 Scans) Frag=135.0V XCI_50_6_TIC.d Subtract
670.4
353.4
Counts vs. Mass-to-Charge (m/z)

5f

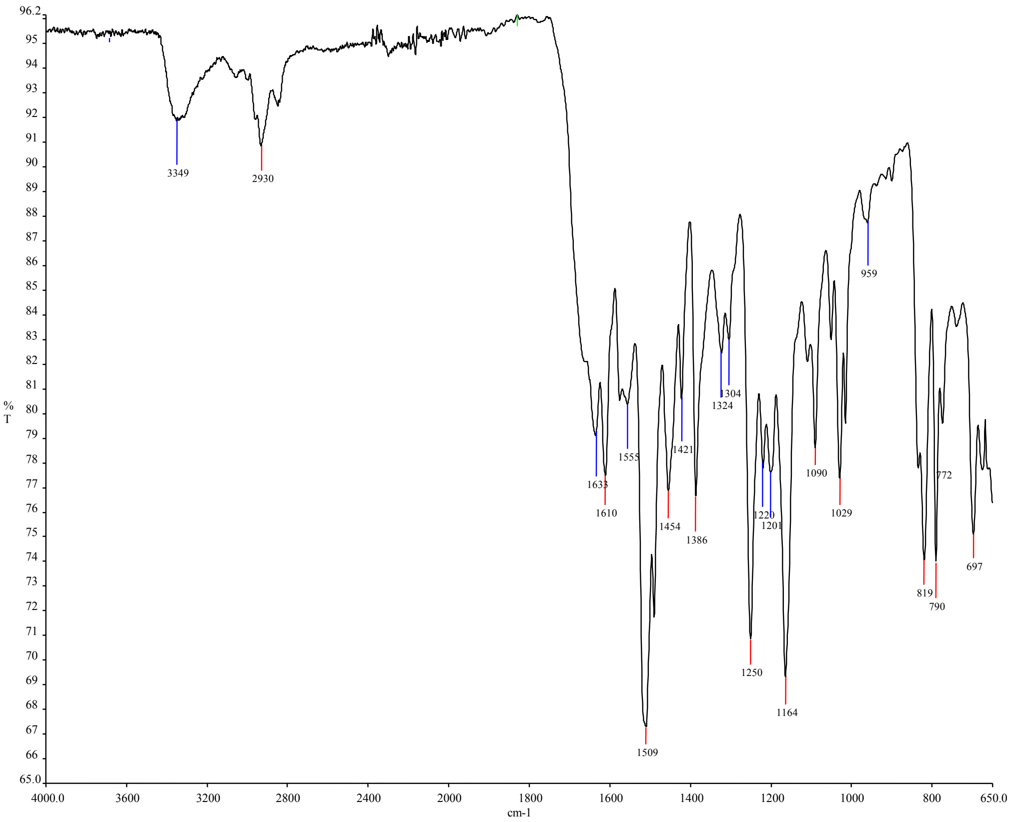
%T
cm-1
3349
2930
1633
1610
1555
1509
1454
1421
1386
1324
1304
1250
1220
1201
1164
1090
1029
959
819
790
772
697

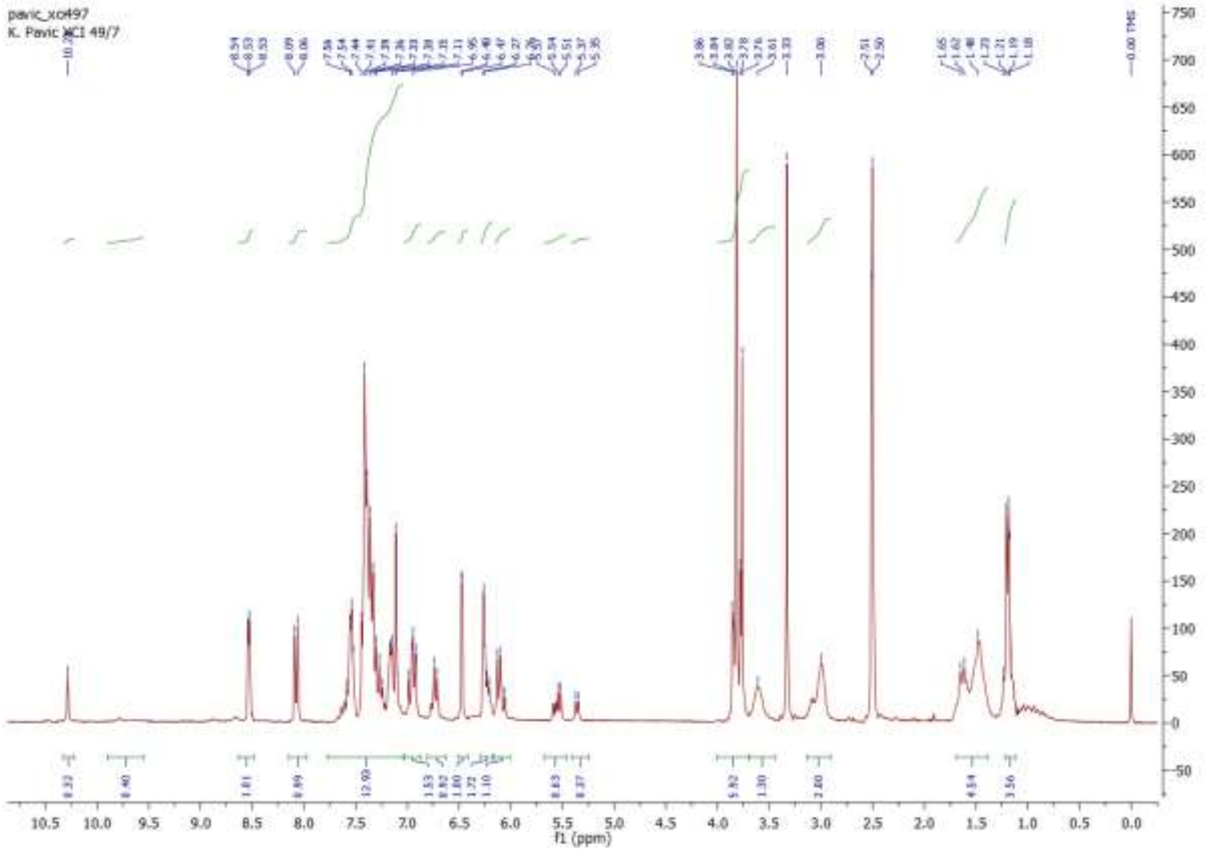
pavic_xc497
f1 (ppm)

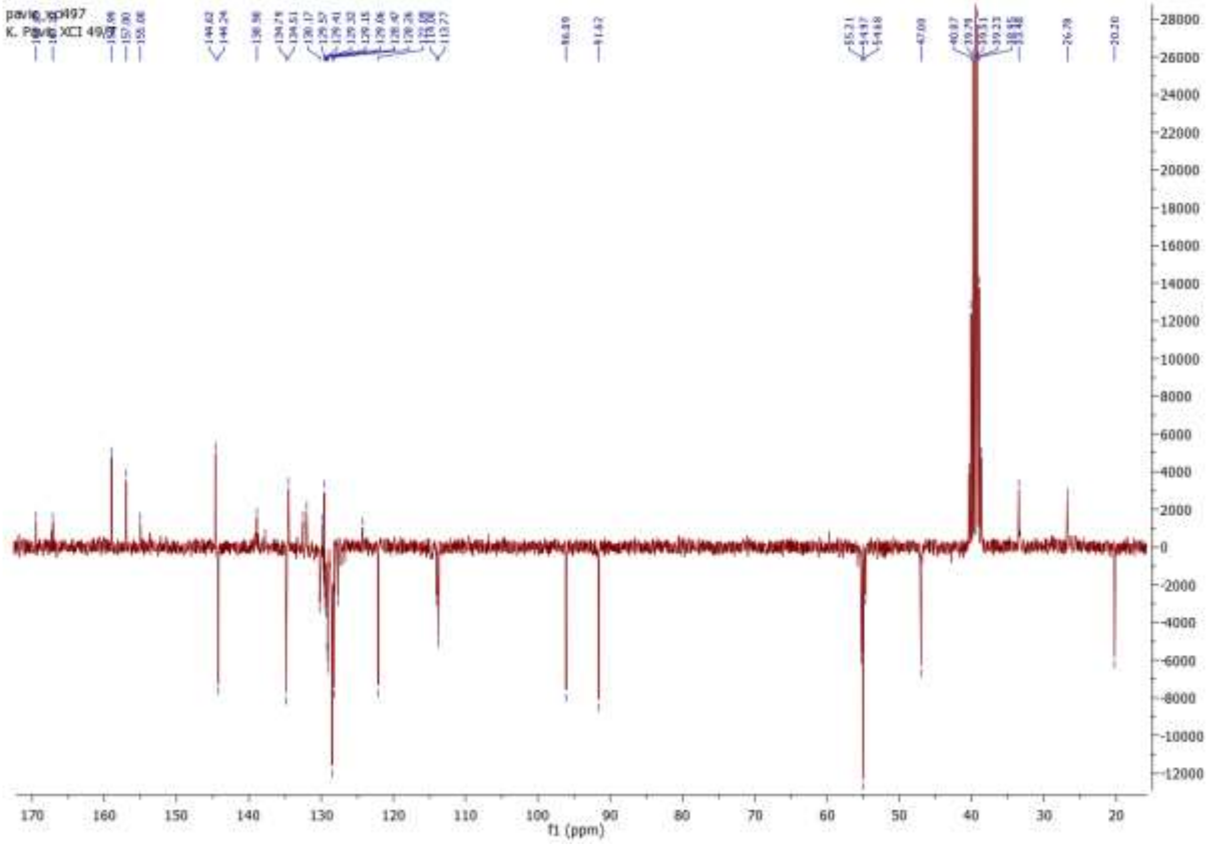
f1 (ppm)

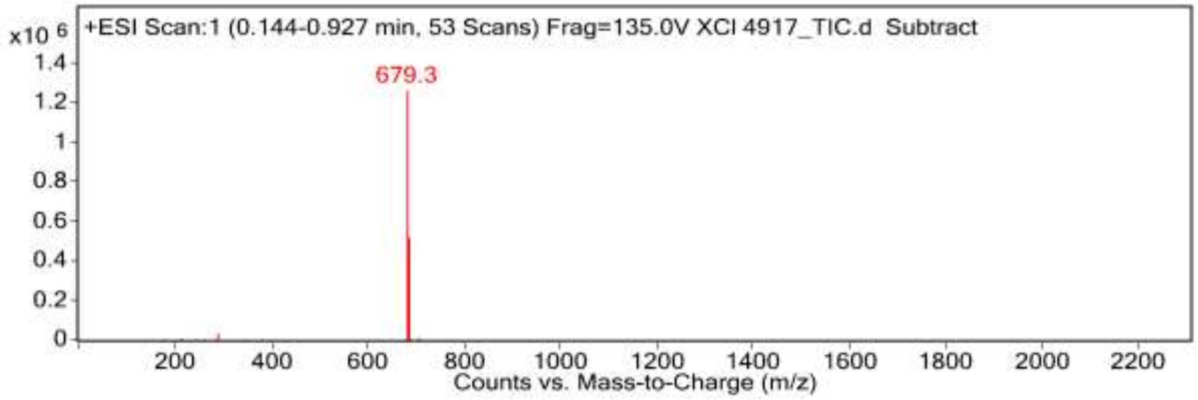
+ESI Scan:1 (0.144-0.927 min, 53 Scans) Frag=135.0V XCI 4917_TIC.d Subtract
679.3
Counts vs. Mass-to-Charge (m/z)

3. *In vitro* antimicrobial susceptibility assay (MIC determination).

Microorganism	MIC (µg/ml)								
	PQ	**5a**	**5b**	**5c**	**5d**	**5e**	**5f**	TC	Amph
Staphylococcus aureus ATCC 25213	70	6.5	60	>100	12.5	>100	>100	0.16	na
Staphylococcus aureus ATCC 6538	50	12.5	>100	>100	25	>100	>100	0.31	na
Staphylococcus aureus ATCC 25923	45	15	65	>100	25	>100	>100	0.31	na
Enterococcus faecalis ATCC 29212	>100	>100	>100	>100	>100	>100	>100	0.31	na
Enterococcus hyrae ATCC 8043	>100	>100	>100	>100	>100	>100	>100	0.63	na
Kocuria rhizophila ATCC 9341	50	25	>100	>100	>100	>100	>100	0.63	na
Bacillus cereus ATCC 11778	70	70	>100	>100	50	>100	>100	0.31	na
Bacillus subtilis ATCC 6633	80	50	>100	>100	65	>100	>100	0.31	na
Escherichia coli ATCC 8739	40	>100	>100	60	65	60	>100	2.5	na
Escherichia coli ATCC 10536	65	>100	>100	60	60	45	>100	2.5	na
Pseudomonas aeruginosa ATCC 27853	65	25	>100	25	50	>100	>100	3	na
Pseudomonas aeruginosa ATCC 9027	70	60	>100	50	65	>100	>100	3	na
Burkholderia caepacia ATCC 25416	80	65	>100	70	>100	>100	>100	3	na
Candida albicans ATCC 10231	90	>100	>100	>100	>100	>100	>100	Na	0.5
Candida albicans ATCC 90028	>100	>100	>100	>100	>100	>100	>100	na	0.5
Candida kefyr ATCC 2512	40	>100	>100	>100	>100	>100	>100	na	0.5
Candida krusei ATCC 14243	>100	>100	>100	>100	>100	>100	>100	na	0.5
Candida parapsylosis ATCC 22019	25	>100	>100	>100	>100	>100	>100	6.13	na
Candida tropicalis ATCC 750	>100	>100	>100	>100	>100	>100	>100	na	0.5
Issatchenkia orientalis Kudrjanzev ATCC 6258	>100	>100	>100	>100	>100	>100	>100	na	0.5
Aspergillus niger ATCC 16404	>100	>100	>100	>100	>100	>100	>100	na	0.5

na – no activity; TC – tetracycline; Amph – amphotericin B.

4. Reference

1. Butula I, Zorc B, Vela V. Reaktionen mit 1-Benzotriazol carbonsaurechlorid.VII. Die Umsetzung mit Aminosauren. Croat Chem Acta, 1981;54:435–40.
